# Cardiovascular outcomes in trials of new antidiabetic drug classes: a network meta-analysis

**DOI:** 10.1186/s12933-019-0916-z

**Published:** 2019-08-28

**Authors:** Yue Fei, Man-Fung Tsoi, Bernard Man Yung Cheung

**Affiliations:** 1 Division of Clinical Pharmacology and Therapeutics, Department of Medicine, The University of Hong Kong, Queen Mary Hospital, 102 Pokfulam Road, Pokfulam, Hong Kong, China; 2 State Key Laboratory of Pharmaceutical Biotechnology, The University of Hong Kong, Pokfulam, Hong Kong, China; 3 Institute of Cardiovascular Science and Medicine, The University of Hong Kong, Pokfulam, Hong Kong, China

**Keywords:** Antidiabetic drug, Network meta-analysis, Type 2 diabetes mellitus, Cardiovascular outcome

## Abstract

**Background:**

Recent trials suggested that glucagon-like peptide-1 receptor agonists (GLP-1 RAs) and sodium-glucose co-transporter-2 (SGLT-2) inhibitors reduced cardiovascular events. Comparative effectiveness of these new antidiabetic drug classes remains unclear. We therefore performed a network meta-analysis to compare the effect on cardiovascular outcomes among GLP-1 RAs, SGLT-2 and dipeptidyl peptidase-4 (DPP-4) inhibitors.

**Methods:**

MEDLINE, EMBASE, Cochrane database, ClinicalTrials.gov, and congress proceedings from recent cardiology conferences were searched up to April 20, 2019. Cardiovascular outcome trials and renal outcome trials reporting cardiovascular outcomes on GLP-1 RAs, SGLT-2 and DPP-4 inhibitors in patients with type 2 diabetes mellitus were included. The primary outcome was major adverse cardiovascular events (MACE). Secondary outcomes were nonfatal myocardial infarction, nonfatal stroke, cardiovascular mortality, all-cause mortality, hospitalisation for heart failure (HF), and renal composite outcome. ORs and 95% CI were calculated using random-effects models.

**Results:**

Fourteen trials enrolling 121,047 patients were included. SGLT-2 inhibitors reduced cardiovascular deaths and all-cause deaths compared to placebo (OR 0.82, 95% CI 0.73–0.93 and OR 0.84, 95% CI 0.77–0.92) and DPP-4 inhibitors (OR 0.83, 95% CI 0.70–0.99 and OR 0.83, 95% CI 0.73–0.94), respectively. SGLT-2 inhibitors and GLP-1 RAs significantly reduced MACE (OR 0.88, 95% CI 0.82–0.95 and OR 0.87, 95% CI 0.82–0.93), hospitalisation for HF (OR 0.68, 95% CI 0.61–0.77 and OR 0.87, 95% CI 0.82–0.93), and renal composite outcome (OR 0.59, 95% CI 0.52–0.67 and OR 0.86, 95% CI 0.78–0.94) compared to placebo, but SGLT-2 inhibitors reduced hospitalisation for HF (OR 0.79, 95% CI 0.69–0.90) and renal composite outcome (OR 0.69, 95% CI 0.59–0.80) more than GLP-1 RAs. Only GLP-1 RAs reduced nonfatal stroke (OR 0.88, 95% CI 0.77–0.99). DPP-4 inhibitors did not lower the risk of these outcomes when compared to placebo and were associated with higher risks of MACE, hospitalisation for HF, and renal composite outcome when compared to the other two drug classes.

**Conclusions:**

SGLT-2 inhibitors show clear superiority in reducing cardiovascular and all-cause deaths, hospitalisation for HF, and renal events among new antidiabetic drug classes. GLP-1 RAs also have cardiovascular and renal protective effects. DPP-4 inhibitors have no beneficial cardiovascular effects and are therefore inferior to the other two drug classes. SGLT-2 inhibitors should now be the preferred treatment for type 2 diabetes mellitus.

## Background

Type 2 diabetes mellitus (T2DM) is rising in prevalence and is a major cause of morbidity and mortality worldwide [1]. Cardiovascular disease, especially myocardial infarction (MI) and stroke, is the primary cause of complications and deaths in patients with T2DM [2]. Prevention of cardiovascular disease is, therefore, a goal of treatment of T2DM as important as glycaemic control. Metformin is the first-line therapy according to the American Diabetes Association (ADA)/European Association for the Study of Diabetes (EASD) [3] and the International Diabetes Federation [4]. However, metformin is contraindicated or not tolerated in some patients [5]. Rosiglitazone, another class of antidiabetic drug, was withdrawn due to increased cardiovascular events, which prompted the US Food and Drug Administration and the European Medicines Agency to require all new antidiabetic drugs to undergo large cardiovascular outcome trials (CVOTs) to rule out excess cardiovascular risk [6, 7].

In recent years, new antidiabetic drug classes, such as glucagon-like peptide 1 (GLP-1) receptor agonists (RAs), sodium-glucose co-transporter-2 (SGLT-2) inhibitors, and dipeptidyl peptidase-4 (DPP-4) inhibitors, with low risk of hypoglycaemia and weight gain, have become available and are now widely used as add-on therapies after metformin [3, 4, 8]. Their cardiovascular benefits have been reported in a number of studies. In the Liraglutide Effect and Action in Diabetes: Evaluation of Cardiovascular Outcome Results (LEADER) and the Empagliflozin Cardiovascular Outcome Event Trial in Type 2 Diabetes Mellitus Patients (EMPA-REG OUTCOME) trials, liraglutide and empagliflozin were found to decrease cardiovascular events, cardiovascular mortality, and all-cause mortality, respectively [9, 10]. These benefits were confirmed in our previous meta-analysis that included these two CVOTs [11] and supported the guidelines recommending these two drug classes for T2DM patients with arteriosclerotic cardiovascular disease [3, 4, 8]. Recently, more CVOTs including Harmony Outcomes [12] and Exenatide Study of Cardiovascular Event Lowering (EXSCEL) [13] on GLP-1 RAs, Dapagliflozin Effect on Cardiovascular Events-Thrombolysis in Myocardial Infarction (DECLARE-TIMI) 58 [14], CANaglifozin cardioVascular Assessment Study (CANVAS) programs [15] on SGLT-2 inhibitors, and Cardiovascular and Renal Microvascular Outcome Study With Linagliptin (CARMELINA) [16] on a DPP-4 inhibitor have been completed. The findings of EXSCEL [13] and CANVAS programs [15] have not only confirmed the cardiovascular safety profiles of GLP-1 RAs and SGLT-2 inhibitors but also affected the recommendations on the personalised diabetes management in the ADA/EASD consensus statement [3]. The other CVOTs reported after the publication of the ADA/EASD consensus statement showed inconsistent results compared with previous evidence. The superiority of albiglutide, a GLP-1 RA in reducing major adverse cardiovascular events (MACE) was found in Harmony Outcomes (P = 0.006) [12]. However, there was no significant reduction in MACE with dapagliflozin, a SGLT-2 inhibitor (P = 0.17) in DECLARE-TIMI 58 [14]. A recent meta-analysis including these two latest CVOTs showed a similar protection of GLP-1 RAs and SGLT-2 inhibitors against MACE [17], although DPP-4 inhibitors, a widely-used antidiabetic drug class, were not evaluated.

Like cardiovascular disease, kidney disease is also a common complication in diabetic patients and is associated with increased morbidity and mortality. It has been suggested that GLP-1 RAs and SGLT-2 inhibitors might improve renal outcomes in patients with T2DM [17]. Recently, the first renal outcome trial on SGLT-2 inhibitors, Canagliflozin and Renal Endpoints in Diabetes with Established Nephropathy Clinical Evaluation (CREDENCE), has just been reported, showing promising renal and cardiovascular benefits [18].

Nevertheless, uncertainty remains as to whether a specific antidiabetic drug class is superior to others in reducing cardiovascular events, due to the lack of direct comparative CVOTs between the new antidiabetic drug classes with sufficient power. Moreover, it is uncertain whether these newly published trials necessitate an update of current guidelines. Therefore, we used network meta-analysis to simultaneously compare all the new antidiabetic drug classes with respect to mortality and cardiovascular outcomes.

## Methods

This network meta-analysis was reported in accordance with the Preferred Reporting Items for Systematic reviews and Meta-Analyses (PRISMA) Statement. The protocol was registered with the PROSPERO registry (number CRD42016050146).

### Search strategy

MEDLINE, EMBASE, the Cochrane database, ClinicalTrials.gov, and congress proceedings from recent cardiology conferences were searched up to April 20, 2019 without applying any language and publication status restrictions. Our search threads were limited to large outcome trials evaluating new antidiabetic drug classes, namely, GLP-1 RAs, SGLT-2 or DPP-4 inhibitors in patients with T2DM. The search keywords included “glucagon-like peptide-1 receptor agonist”, “sodium-glucose co-transporter 2 inhibitor”, “dipeptidyl peptidase-4 inhibitor”, “major adverse cardiovascular event (MACE)”, “cardiovascular risk”, “cardiovascular event”, “type 2 diabetes mellitus”, and their synonyms and related keywords.

### Study selection

Studies were eligible to be included if they met the following inclusion criteria for this network meta-analysis: (1) phase III/IV CVOTs or renal outcome trials evaluating cardiovascular outcomes; (2) allocation of GLP-1 RAs, SGLT-2 or DPP-4 inhibitors; (3) patients with T2DM (≥ 18 years of age); (4) patients with established cardiovascular disease or cardiovascular risk factors; (5) report of the rate of MACE and deaths.

### Data extraction

Two investigators (YF and MFT) independently performed literature search and selection. Discrepancies were resolved by consensus. For eligible studies, information about the year of publication, age, gender, sample size, body weight, ethnic group, smoking status, intervention, duration of follow-up, glycated haemoglobin, baseline comorbidities of cardiovascular disease or cardiovascular risk factors, duration of diabetes, and duration of follow-up were extracted. Quality of the included studies and risk of trial bias were assessed using the Cochrane risk of bias assessment tool.

### Study outcomes

The primary outcome in our network meta-analysis was MACE, defined as the composite of cardiovascular mortality, nonfatal MI, and nonfatal stroke. Secondary outcomes included the individual components of the primary outcome, all-cause mortality, hospitalisation for heart failure (HF), and renal composite outcome, defined as a composite of adjudication-confirmed end-stage renal disease, death due to renal failure, or a sustained decrease of at least 40% in estimated glomerular filtration rate (eGFR) from baseline to less than 60 ml per minute per 1.73 m^2^ of body-surface area [19]. Definitions of the renal composite outcome in the included trials are listed in Additional file 1: Table S1. We followed the definitions of outcomes used in each trial.

### Statistical analysis

We used both a frequentist approach [20] and a Bayesian framework [21] with non-informative priors to compare the effect of different antidiabetic drug classes on outcomes at the trial level. Count data were extracted to analyse study outcomes. Pooled random-effects odds ratios (ORs) and 95% confidence intervals (95% CIs) were used as the summary statistics. A 95% CI not including 1.00 or a two-tailed *p* value less than 0.05 was considered statistically significant. Forest plots comparing relative treatment effects were generated with a frequentist approach using the statistical package ‘netmeta’ (version 0.9–8, https://cran.r-project.org/web/packages/netmeta/index.html) in R (version 3.3.2).

Sensitivity analysis was performed using the fixed-effects model to avoid small-study effects and was conducted by assessing the effect of excluding individual trials. Inconsistency in the network was evaluated using loop-specific heterogeneity estimates; τ^2^ estimate of 0.04, 0.14, and 0.40 represented a low, moderate, and high degree of variance, respectively. Heterogeneity within each pairwise meta-analysis of the direct comparisons of antidiabetic drug classes was assessed by I^2^ statistic. I^2^ < 25%, 25% to 50%, and > 50% corresponded to mild, moderate, and severe heterogeneity, respectively. Potential publication bias was assessed using funnel plots, Begg’s, Egger’s, and trim-and-fill tests for direct comparisons with three or more studies.

We evaluated the consistency of inferential estimates from hierarchical modelling using Markov chain Monte Carlo simulations, which were performed with 1000 tuning iterations and 5000 simulation iterations within a Bayesian framework using R statistical package ‘gemtc’ (version 0.8–2, https://cran.r-project.org/web/packages/gemtc/index.html) and ‘rjags’ (version 4–6, https://cran.r-project.org/web/packages/rjags/index.html) to minimise Monte Carlo error. The relative probability of each antidiabetic drug class having the largest effect size on each outcome was generated using P-rank scores.

## Results

The literature search and selection process are shown in the PRISMA flowchart (Additional file 1: Fig. S1). Twenty-four trials initially fulfilled the inclusion criteria [9, 10, 12–16, 18, 22–38]. However, data on MACE and mortality have not yet been reported for the following ongoing trials: CARdiovascular Outcome Trial of LINAgliptin Versus Glimepiride in Type 2 Diabetes (CAROLINA) [29], Vildagliptin in Ventricular Dysfunction Diabetes (VIVIDD) [30], FREEDOM-CVO [31], Researching cardiovascular Events with a Weekly INcretin in Diabetes (REWIND) [32], PIONEER 6 [33], eValuation of ERTugliflozin efficacy and Safety CardioVascular outcomes (VERTIS CV) [34], Dapagliflozin And Prevention of Adverse-outcomes in Heart Failure (Dapa-HF) [35], A Study to Evaluate the Effect of Dapagliflozin on Renal Outcomes and Cardiovascular Mortality in Patients With Chronic Kidney Disease (DAPA CKD) [36], EMPagliflozin outcomE tRial in Patients With chrOnic heaRt Failure With Preserved Ejection Fraction (EMPEROR-Preserved) [37], and EMPagliflozin outcomE tRial in Patients With chrOnic heaRt Failure With Reduced Ejection Fraction (EMPEROR-Reduced) [38], so they were all excluded. Finally, thirteen two-armed CVOTs [9, 10, 12–16, 22–28] and one renal outcome trial [18] with altogether 121,047 patients were eligible for this network meta-analysis.

**Fig. 1:**
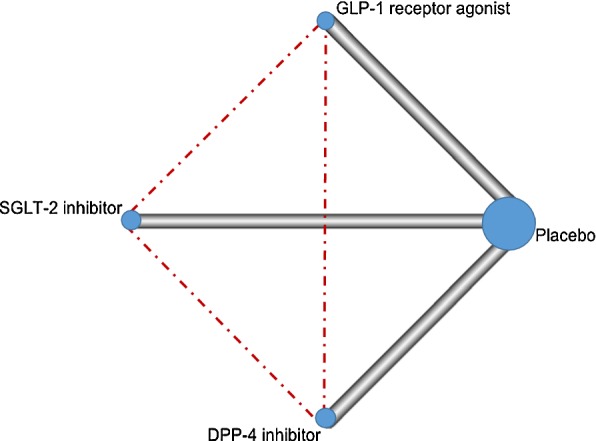
Network profile for the included trials comparing different antidiabetic drug classes. Each line represents a pair of direct comparison between different antidiabetic drug classes while each dotted line represents the missing comparison. The width of the lines is proportional to the number of trials comparing every pair of treatments, and the size of every circle is proportional to the number of randomly assigned participants (sample size)

There were five trials including Evaluation of Lixisenatide in Acute Coronary Syndrome (ELIXA), LEADER, Trial to Evaluate Cardiovascular and Other Long-term Outcomes with Semaglutide in Subjects with Type 2 Diabetes (SUSTAIN-6), Harmony Outcomes, and EXSCEL [9, 12, 13, 22, 24, 27] in the comparison between GLP-1 RAs vs. placebo, five trials including EMPA-REG OUTCOME, CANVAS, CANVAS-R, DECLARE-TIMI 58, and CREDENCE [10, 14, 15, 18, 28] in the comparison between SGLT-2 inhibitors vs. placebo, and four trials including Saxagliptin Assessment of Vascular Outcomes Recorded in Patients with Diabetes Mellitus (SAVOR)-Thrombolysis in Myocardial Infarction (TIMI) 53, Examination of Cardiovascular Outcomes with Alogliptin versus Standard of Care (EXAMINE), Trial Evaluating Cardiovascular Outcomes with Sitagliptin (TECOS), and CARMELINA [16, 23, 25, 26] in the comparisons between DPP-4 inhibitors vs. placebo. No direct comparative trials among the three new antidiabetic drug classes were found. Three antidiabetic drug classes and placebo resulted in six theoretical comparisons for each outcome of interest (Fig. 1). The main characteristics of the included trials are listed in Table 1. All the included trials were at low risk of bias (Additional file 1: Tables S2 and S3). Baseline patient characteristics in each trial are shown in Additional file 1: Table S4.

**Table 1:** Major characteristics of the outcome trials included in the network meta-analysis

Studies	Year	ClinicalTrials.gov Identifier	Intervention	Patients (Antidiabetic drug/placebo)	Primary endpoints	HR^a^ (95% CI) of MACE^b^	HR (95% CI) of all-cause mortality
GLP-1 RA vs. placebo							
ELIXA [24]	2015	NCT01147250	Lixisenatide vs. placebo	6068 (3034/3034)	A composite of the first occurrence of any of the following: death from cardiovascular causes, nonfatal MI, nonfatal stroke, or hospitalisation for unstable angina	1.02 (0.89–1.17)	0.94 (0.78–1.13)
LEADER [9]	2016	NCT01179048	Liraglutide vs. placebo	9340 (4668/4672)	A composite of the first occurrence of death from cardiovascular causes, nonfatal MI (including silent), or nonfatal stroke	0.87 (0.78–0.97)	0.85 (0.74–0.97)
SUSTAIN-6 [22]	2016	NCT01720446	Semaglutide vs. placebo	3297 (1648/1649)	A composite of the first occurrence of death from cardiovascular causes, nonfatal MI (including silent), or nonfatal stroke	0.74 (0.58–0.95)	1.05 (0.74–1.50)
HARMONY OUTCOMES [12]	2018	NCT02465515	Albiglutide vs. placebo	9463 (4731/4732)	A composite of death from cardiovascular causes, MI, and stroke	0.78 (0.68–0.90)	0.95 (0.79–1.16)
EXSCEL [13, 27]	2018	NCT01144338	Exenatide vs. placebo	10,782 (5394/5388)	A composite of death from cardiovascular causes, nonfatal MI, and nonfatal stroke	0.90 (0.82–1.00)	0.88 (0.77–1.00)
DPP-4 inhibitor vs. placebo							
SAVOR-TIMI 53 [26]	2013	NCT01107886	Saxagliptin vs. placebo	16,492 (8280/8212)	A composite of cardiovascular death, nonfatal MI, or nonfatal ischaemic stroke	1.00 (0.89–1.12)	1.11 (0.96–1.27)
EXAMINE [25]	2013	NCT00968708	Alogliptin vs. placebo	5380 (2701/2679)	A composite of death from cardiovascular causes, nonfatal MI, or nonfatal stroke	0.96 (≤ 1.16)^c^	0.98 (0.86–1.12)
TECOS [23]	2015	NCT00790205	Sitagliptin vs. placebo	14,523 (7257/7266)	A composite of the first confirmed event of cardiovascular death, nonfatal MI, nonfatal stroke, or hospitalisation for unstable angina	0.99 (0.89–1.10)	1.01 (0.90–1.14)
CARMELINA [16]	2018	NCT01897532	Linagliptin vs. placebo	6979 (3494/3485)	A composite of the first occurrence of cardiovascular death, nonfatal MI, or nonfatal stroke	1.02 (0.89–1.17)	0.98 (0.84–1.13)
SGLT-2 inhibitor vs. placebo							
EMPA-REG OUTCOME [10, 28]	2015	NCT01131676	Empagliflozin vs. placebo	7020 (4687/2333)	A composite of death from cardiovascular causes, nonfatal MI (excluding silent MI), or nonfatal stroke	0.86 (0.74–0.99)	0.68 (0.57–0.82)
CANVAS [15]	2017	NCT01032629	Canagliflozin vs. placebo	4330 (2888/1442)	A composite of death from cardiovascular causes, nonfatal MI, or nonfatal stroke	0.88 (0.75–1.03)	0.84 (0.70–1.01)
CANVAS-R [15]	2017	NCT01989754	Canagliflozin vs. placebo	5812 (2907/2905)	A composite of death from cardiovascular causes, nonfatal MI, or nonfatal stroke	0.82 (0.66–1.01)	0.92 (0.70–1.21)
DECLARE-TIMI 58 [14]	2018	NCT01730534	Dapagliflozin vs. placebo	17,160 (8582/8578)	A composite of cardiovascular death, MI, or ischaemic stroke	0.93 (0.84–1.03)	0.93 (0.82–1.04)
CREDENCE [18]	2019	NCT02065791	Canagliflozin vs. placebo	4401 (2202/2199)	A composite of cardiovascular death, MI, or stroke	0.80 (0.67–0.95)	0.83 (0.68–1.02)

DPP-4: dipeptidyl peptidase-4; GLP-1: glucagon-like peptide-1; HR: hazard ratio; MACE: major adverse cardiovascular events; MI: myocardial infarction; RA: receptor agonist; SGLT-2: sodium-glucose co-transporter 2; 95% CI: 95% confidence interval

^a^HR (antidiabetic drug vs. placebo) of the outcomes evaluated in each trial

^b^MACE was defined as the composite of nonfatal myocardial infarction, nonfatal stroke, and cardiovascular mortality

^c^Only upper bound of the one-sided 95% CI was reported (α: 0.01)

**Fig. 2:**
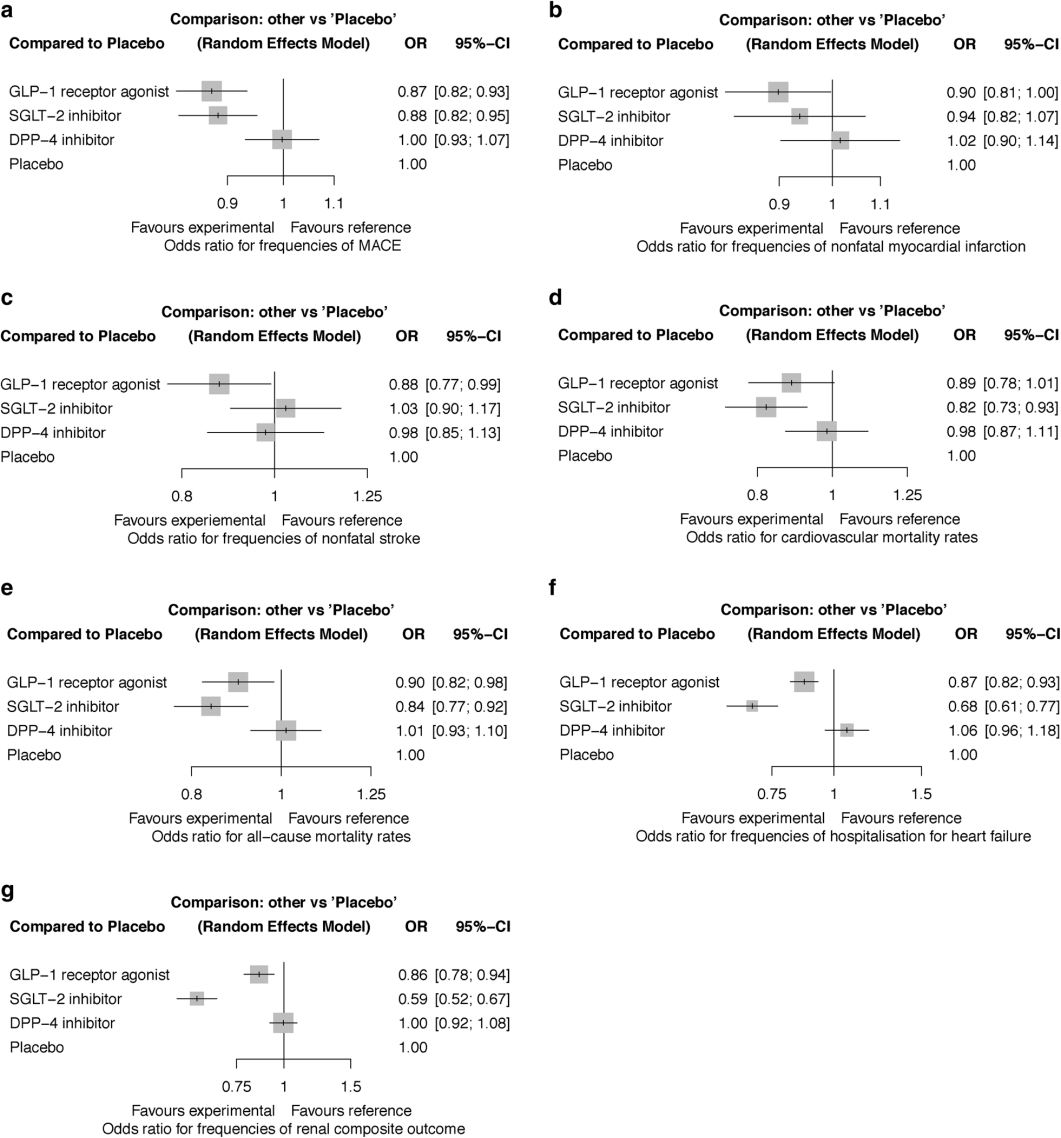
Risk of outcomes with different antidiabetic drug classes compared to placebo. **a** MACE (major adverse cardiovascular events). **b** Nonfatal myocardial infarction. **c** Nonfatal stroke. **d** Cardiovascular mortality. **e** All-cause mortality. **f** Hospitalisation for heart failure. **g** Renal composite outcome

SGLT-2 inhibitors and GLP-1 RAs both significantly lowered the risk of MACE, hospitalisation for HF, and renal composite outcome when compared to placebo and DPP-4 inhibitors, respectively (Table 2 and Fig. 2). SGLT-2 inhibitors reduced hospitalisation for HF and the renal composite outcome more than GLP-1 RAs. Both SGLT-2 inhibitors and GLP-1 RAs reduced all-cause mortality compared to placebo. SGLT-2 inhibitors, moreover, reduced all-cause mortality compared to DPP-4 inhibitors. SGLT-2 inhibitors also resulted in lower cardiovascular mortality than both placebo and DPP-4 inhibitors (Table 2). In addition, GLP-1 RAs recipients had fewer nonfatal strokes than those receiving placebo. There was a trend towards a lower risk of nonfatal MI with GLP-1 RAs when compared to placebo, although the significance of result was marginal (p = 0.044). In contrast, DPP-4 inhibitors showed a similar risk profile to placebo in all the outcomes (Fig. 2).

**Table 2:** Network meta-analysis of efficacy and safety outcomes of new antidiabetic drug classes using random-effects model

MACE			
GLP-1 RA	1.01 (0.92–1.12)	*1.14 (1.04–1.26)*	*1.14 (1.07–1.22)*
0.99 (0.89–1.09)	SGLT-2 inhibitor	*1.13 (1.02–1.25)*	*1.13 (1.05–1.22)*
*0.88 (0.80–0.96)*	*0.89 (0.80–0.98)*	DPP-4 inhibitor	1.00 (0.94–1.07)
*0.87 (0.82–0.93)*	*0.88 (0.82–0.95)*	1.00 (0.93–1.07)	Placebo
Nonfatal myocardial infarction			
GLP-1 RA	1.04 (0.88–1.23)	1.13 (0.97–1.32)	*1.11 (1.00–1.24)*
0.96 (0.81–1.13)	SGLT-2 inhibitor	1.08 (0.91–1.29)	1.07 (0.94–1.22)
0.88 (0.78–1.04)	0.92 (0.77–1.10)	DPP-4 inhibitor	0.98 (0.88–1.11)
*0.90 (0.81–1.00)*	0.94 (0.82–1.07)	1.02 (0.90–1.14)	Placebo
Nonfatal stroke			
GLP-1 RA	1.17 (0.98–1.41)	1.12 (0.93–1.35)	*1.14 (1.01–1.29)*
0.85 (0.71–1.02)	SGLT-2 inhibitor	0.95 (0.79–1.16)	0.97 (0.85–1.11)
0.89 (0.74–1.08)	1.05 (0.87–1.27)	DPP-4 inhibitor	1.02 (0.89–1.18)
*0.88 (0.77–0.99)*	1.03 (0.90–1.17)	0.98 (0.85–1.13)	Placebo
Cardiovascular mortality			
GLP-1 RA	0.93 (0.78–1.11)	1.11 (0.93–1.33)	1.13 (0.99–1.28)
1.08 (0.90–1.29)	SGLT-2 inhibitor	*1.20 (1.01–1.42)*	*1.22 (1.08–1.38)*
0.90 (0.75–1.07)	*0.83 (0.70–0.99)*	DPP-4 inhibitor	1.02 (0.90–1.15)
0.89 (0.78–1.01)	*0.82 (0.73–0.93)*	0.98 (0.87–1.11)	Placebo
All-cause mortality			
GLP-1 RA	0.93 (0.82–1.06)	1.13 (0.99–1.28)	*1.11 (1.02–1.22)*
1.07 (0.94–1.22)	SGLT-2 inhibitor	*1.20 (1.06–1.37)*	*1.19 (1.09–1.30)*
0.89 (0.78–1.01)	*0.83 (0.73–0.94)*	DPP-4 inhibitor	0.99 (0.91–1.08)
*0.90 (0.82–0.99)*	*0.84 (0.77–0.92)*	1.01 (0.93–1.10)	Placebo
Hospitalisation for heart failure			
GLP-1 RA	*0.79 (0.69–0.90)*	*1.22 (1.08–1.37)*	*1.15 (1.08–1.22)*
*1.27 (1.11–1.45)*	SGLT-2 inhibitor	*1.55 (1.33–1.81)*	*1.46 (1.30–1.64)*
*0.82 (0.73–0.92)*	*0.64 (0.55–0.75)*	DPP-4 inhibitor	0.94 (0.85–1.04)
*0.87 (0.82–0.93)*	*0.68 (0.61–0.77)*	1.06 (0.96–1.18)	Placebo
Renal composite outcome			
GLP-1 RA	*0.69 (0.59–0.80)*	*1.16 (1.03–1.31)*	*1.16 (1.06–1.27)*
*1.46 (1.25–1.70)*	SGLT-2 inhibitor	*1.69 (1.46–1.96)*	*1.70 (1.50–1.91)*
*0.86 (0.76–0.98)*	*0.59 (0.51–0.69)*	DPP-4 inhibitor	1.00 (0.92–1.09)
*0.86 (0.78–0.94)*	*0.59 (0.52–0.67)*	1.00 (0.92–1.08)	Placebo

DPP-4: dipeptidyl peptidase-4; GLP-1: glucagon-like peptide-1; MACE: major adverse cardiovascular events (defined as the composite of cardiovascular mortality, nonfatal myocardial infarction and nonfatal stroke); RA: receptor agonist; SGLT-2: sodium-glucose co-transporter 2

Comparisons should be read from left to right. Results are the odds ratios (95% confidence interval) in the column-defining therapy compared with the odds ratios in the row-defining therapy. For efficacy and safety, odds ratio < 1 favours the column-defining therapy. Significant results are shown in italic

P-rank scores confirmed the results of network meta-analysis. SGLT-2 inhibitors were ranked the best in reducing MACE (P = 50.20%), cardiovascular mortality (77.20%), all-cause mortality (82.30%), hospitalisation for HF (99.80%), and renal composite outcome (100.00%). GLP-1 RAs were ranked the second-highest for protecting against these five outcomes while they had the highest likelihood to reduce the risk of nonfatal stroke (80.55%) (Additional file 1: Table S5).

The ELIXA [24] and EXAMINE [25] recruited diabetic patients with acute coronary syndrome within 180 days, which was different from the inclusion criteria in other CVOTs. Sensitivity analysis excluding these two trials increased the significance of the reduction in nonfatal MI with GLP-1 RAs (OR 0.86, 95% CI 0.77–0.97) when compared to placebo. GLP-1 RAs also reduced all-cause deaths more than DPP-4 inhibitors (OR 0.86, 95% CI 0.75–0.98) (Additional file 1: Table S6).

The degree of heterogeneity in this network was not high (Additional file 1: Table S7). In the pairwise meta-analysis, significant heterogeneity was found for MACE (I^2^ = 58%, p = 0.05) in the comparison between GLP-1 RAs vs. placebo, for cardiovascular mortality (I^2^ = 64%, p = 0.03) and all-cause mortality (I^2^ = 50%, p = 0.09) in the comparison between SGLT-2 inhibitors vs. placebo, and for hospitalisation for HF (I^2^ = 54%, p = 0.09) in the comparison between DPP-4 inhibitors vs. placebo. Sensitivity analysis showed that the heterogeneity in the comparison between GLP-1 RAs and placebo was due to the ELIXA trial [24]. Excluding it could reduce I^2^ to 31% (p = 0.23) (Additional file 1: Table S8). The heterogeneity in the comparison between SGLT-2 inhibitors and placebo was due to the EMPA-REG OUTCOME trial [10]. I^2^ could be reduced to 0% after excluding it for cardiovascular mortality (p = 0.51) (Additional file 1: Table S9) and all-cause mortality (p = 0.73) (Additional file 1: Table S10). The heterogeneity in the comparison between DPP-4 inhibitors and placebo was due to the SAVOR-TIMI 53 trial [26]. I^2^ could be reduced to 0% (p = 0.68) after excluding it (Additional file 1: Table S11).

Using fixed-effects instead of random-effects models, or Bayesian instead of frequentist analysis yielded consistent results (Additional file 1: Tables S12 and S13). Inspection of the funnel plots suggested no significant publication bias (Additional file 1: Fig. S2–S8 and Table S14).

## Discussion

Although both GLP-1 RAs and SGLT-2 inhibitors have been reported to reduce cardiovascular events in several trials, which of them yield more cardiovascular benefits has been unclear. Our network meta-analysis including the latest evidence from the CVOTs and renal outcome trial allowed greater statistical power to compare different new antidiabetic drug classes and established their cardiovascular safety profiles. Our findings can be summarised as follows. First, SGLT-2 inhibitors and GLP-1 RAs both lowered the risk of MACE, hospitalisation for HF, and renal events. Second, SGLT-2 inhibitors reduced cardiovascular and all-cause mortality, hospitalisation for HF, and renal events the most among the three antidiabetic drug classes. Third, GLP-1 RA was the only drug class that reduced nonfatal stroke. Finally, DPP-4 inhibitors showed a similar safety profile to placebo and were inferior to GLP-1 RAs and SGLT-2 inhibitors with respect to cardiovascular events and deaths.

### Superiority of SGLT-2 inhibitors

What is new in this network meta-analysis is the confirmation that SGLT-2 inhibitors are now superior to the other antidiabetic drug classes in terms of cardiovascular and renal endpoints, especially deaths, making it arguably the drug class of choice. Our findings on SGLT-2 inhibitors are consistent with recent meta-analyses of CVOTs on this drug class [17, 39], in which SGLT-2 inhibitors were found to reduce MACE, cardiovascular mortality, hospitalisation for HF, and progression of renal disease in patients with atherosclerotic cardiovascular disease. However, through network meta-analysis, we could draw comparisons with other drug classes. By including all the relevant trials completed recently, we extended the findings of previous meta-analyses by ourselves and other researchers [11, 17, 39–45]. The significant benefits of SGLT-2 inhibitors in reducing MACE, hospitalisation for HF, and renal events were not only confirmed when compared to placebo but also identified when compared to the other new antidiabetic drug classes. There are several possible mechanisms behind these benefits. The natriuretic response through SGLT2 inhibition in the proximal tubule and the inhibition of the tubuloglomerular feedback may play an important role in the reduction in hospitalisation for HF and the progression of diabetic kidney disease [46]. The weight loss and reduction in blood pressure may contribute to the cardiovascular protection of SGLT-2 inhibitors [47]. Left ventricular (LV) diastolic dysfunction is strongly associated with HF in T2DM patients. The improved LV diastolic function [48] and reduced tissue sodium content [49] with dapagliflozin lend support to these possible mechanisms. In addition, SGLT-2 inhibitors inhibit the Na +/H + exchanger (NHE) 1 in the myocardium results and NHE3 in the proximal tubule, resulting in decreased cytoplasmic sodium and calcium levels while increasing mitochondrial calcium level and inhibiting sodium reabsorption, all of which help to explain the reduction in HF with this drug class [46, 50]. The cardiac antifibrotic effects of SGLT-2 inhibitors found in rat models with dapagliflozin [51] and in human cardiac fibroblasts with empagliflozin [52] may also explain the protective effect of SGLT-2 inhibitors on HF.

DECLARE-TIMI 58 [14] confirmed the benefit of reduction in HF hospitalisation with SGLT-2 inhibitors, but did not show a significantly lower rate of MACE which contrasted with the findings from the EMPA-REG OUTCOME [10], CANVAS [15] and CANVAS-R [15] trials (Table 1, Additional file 1: Table S15). The differences in patient characteristics and drug used across trials could be possible explanations. More than half of the T2DM patients enrolled in DECLARE-TIMI 58 did not have cardiovascular disease but only had risk factors. On the contrary, almost all the patients included in EMPA-REG OUTCOME and more than half of the patients included in CANVAS and CANVAS-R were T2DM patients with established cardiovascular disease. It was more difficult to demonstrate a reduction in MACE in patients without pre-existing cardiovascular disease in DECLARE-TIMI 58. It is possible that dapagliflozin used in this trial has less protection against MACE compared to empagliflozin and canagliflozin. Nevertheless, the reduction in HF hospitalisation with dapagliflozin was consistent across multiple subgroups regardless of history of atherosclerotic cardiovascular disease, HF and CKD. This was also found in other studies of dapagliflozin in humans [48, 49], in animals [51], and in vitro [52]. These diverse pieces of evidence point to the specific protection of HF by dapagliflozin.

All the CVOTs on SGLT-2 inhibitors except EMPA-REG OUTCOME [28] showed robust reductions in renal composite outcomes regardless of baseline factors. The renal benefits of SGLT-2 inhibitors were further confirmed in our network meta-analysis after including CREDENCE [18], the first renal outcome trial. Now the application of SGLT-2 inhibitors can be expanded to T2DM populations with CKD in addition to cardiovascular diseases. The renal protection of SGLT-2 inhibitors needs to be further confirmed in the ongoing renal outcome trial DAPA CKD [36], which recruited CKD patients with or without diabetes. This trial together with CREDENCE will answer whether SGLT-2 inhibitors could be considered a promising option for renal protection in either T2DM or nondiabetic patients.

Most importantly, SGLT-2 inhibitors were found to be significantly better in reducing cardiovascular and all-cause mortality regardless of existing cardiovascular disease or CKD in patients with T2DM. The superiority of SGLT-2 inhibitors over GLP-1 RAs and DPP-4 inhibitors in reducing deaths can, therefore, be considered a class effect. However, the difference in overall mortality did not reach statistical significance (OR 0.83, 95% CI 0.68–1.02) in the CREDENCE trial [18], as with other trials on SGLT-2 inhibitors [14, 15], which differed from the findings in the EMPA-REG OUTCOME trial [10] and this network meta-analysis. Despite the high rate of drug discontinuation (27% of participants), the CREDENCE trial was stopped early because of the overwhelming benefits in lowering the risk of kidney failure and cardiovascular events. As a result of the shortened follow-up period, the study lacked power to demonstrate any mortality reduction. In contrast, EMPA-REG OUTCOME showed a reduction in mortality, so there was heterogeneity in this outcome, although the indirect evidence also supported a reduction in mortality. The protection against deaths observed with empagliflozin but not the other SGLT-2 inhibitors needs to be further explored in future trials to understand whether it is a specific effect of empagliflozin.

### GLP-1 RAs and DPP-4 inhibitors

The favourable cardiovascular safety of GLP-1 RAs, especially their positive effects in reducing MACE, nonfatal stroke, and nonfatal MI, were revealed in our network meta-analysis after including the latest evidence. The reported significant decrease in cardiovascular and all-cause mortality in GLP-1 RA recipients in the LEADER trial [9] was not found in our network meta-analysis or other CVOTs on GLP-1 RAs. Although the improvement in cardiovascular mortality was reported in a recent meta-analysis of the CVOTs on GLP-1 RAs, Harmony Outcomes trial was not included while the results were largely driven by the LEADER trial [53]. The shorter duration of CVOTs besides LEADER reduced their power to show an effect on cardiovascular deaths. Moreover, trial results are also affected by patient characteristics and the different GLP-1 RAs used across the trials.

Liraglutide reduces postprandial glucose without increasing insulin concentration and improves beta-cell function in T2DM patients [54]. Its cardiovascular protective effects have been studied. Reduction in arterial stiffness, LV myocardial strain, twisting and untwisting, N-terminal pro-brain natriuretic peptide, and oxidative stress with liraglutide could offer myocardial protection and prevention of diabetic heart disease [55]. Moreover, liraglutide reduces postprandial non-esterified free fatty acids and suppresses soluble vascular cell adhesion molecule-1 compared to metformin [56]. It protects against acute liver injury in the mouse [57]. However, cardiovascular benefits have not been observed in studies of exenatide [13, 58]. In low-risk patients undergoing scheduled coronary artery bypass grafting surgery, postoperative intravenous exenatide provided no additional cardio-protective effect compared to intravenous insulin [58]. Therefore, it is still necessary to study the cardiovascular efficacy and safety profile of GLP-1 RAs so as to confirm their place in clinical practice.

There are several ongoing CVOTs evaluating the efficacy and safety of GLP-1 RAs in different patient populations with different drug formulations. The FREEDOM-CVO trial is the first trial studying ITCA 650, the first injection-free GLP-1 RA with a full year of treatment delivery from a single placement of an osmotic mini-pump [31]. In the REWIND study on dulaglutide, 70% of patients did not have established cardiovascular disease at baseline [32]. The PIONEER 6 trial investigating oral semaglutide recently announced an insignificant reduction in MACE but a significantly lower risk of cardiovascular and all-cause deaths [33]. These trials will add to the evidence on the cardiovascular effects of GLP-1 RAs, and clarify their effects on mortality.

The cardiovascular benefits observed in GLP-1 RAs were not found in another incretin-based drug class, namely DPP-4 inhibitors. Our network meta-analysis including the latest evidence from CARMELINA [16] still failed to demonstrate cardiovascular benefits with DPP-4 inhibitors, like previous meta-analyses [11, 41, 59–61]. Although a few meta-analyses reported substantial reductions in MACE with DPP-4 inhibitors [62, 63], their conclusions were based on studies with small sample sizes and limited numbers of cardiovascular events, which might be underpowered.

Although no increase in the risk of cardiovascular events was found with DPP-4 inhibitors, a red flag was raised in terms of increased HF. Saxagliptin was observed to result in a significant increase in hospitalisations for HF in the SAVOR-TIMI 53 trial [26]. Alogliptin in the EXAMINE trial [25] showed a trend toward an increased risk for HF hospitalisation while sitagliptin in the TECOS trial showed no increased risk [23]. In the latest CARMELINA, no increase in HF hospitalisation and renal composite outcome, and a significant reduction in microvascular events driven primarily by the reduction in albuminuria progression (p = 0.003) were found with linagliptin. It established the cardiovascular safety profile of linagliptin in diabetic patients at high risk of cardiovascular and kidney disease, those most vulnerable to developing HF. The conflicting effects of DPP-4 inhibitors on hospitalisation for HF need to be reconciled when more evidence becomes available.

In our study, GLP-1 RAs were better than DPP-4 inhibitors in reducing hospitalisation for HF. A retrospective cohort study of T2DM patients reported a significant reduction in HF with DPP-4 inhibitors compared to GLP-1 RAs. The association was consistent in patients with or without prior cardiovascular disease, but not statistically significant in those with prior HF [64]. The DPP-4 inhibitors used in this study were saxagliptin and sitagliptin, while the GLP-1 RAs compared were not specified. Whether the association was due to different effects within the same drug class remained unclear. Besides, the superiority of GLP-1 RAs to DPP-4 inhibitors found in our network meta-analysis was driven largely by indirect evidence due to the absence of head-to-head trials between these two classes. Until such evidence is available, observational studies provide real-world evidence of efficacy.

The two incretin-based drug classes, DPP-4 inhibitors and GLP-1 RAs have different effects on cardiovascular outcomes. GLP-1 RAs directly activate GLP-1 receptors in the arteries and kidneys. The improved endothelial function through nitric oxide-induced vasodilation and the reduced oxidative stress and natriuretic effect by inhibiting the renin–angiotensin–aldosterone system all confer their effects on blood pressure lowering. Also, GLP-1 RAs could protect against endothelial dysfunction and have direct effects on the myocardium, which might also account for their beneficial effects in reducing vascular disease [65].

### Clinical implications

A retrospective cohort study on second-line antidiabetic drugs showing similar efficacy and safety profiles of GLP-1 RAs, SGLT-2 and DPP-4 inhibitors on cardiovascular events has been published [66]. However, observational studies may be prone to selection bias. The imbalance of the patient numbers in each drug class, in which DPP-4 inhibitors recipients (28,898) were five times more than SGLT-2 inhibitors recipients (5677) and twice more than GLP-1 RAs recipients (11,351) limited its statistical power without using propensity score matching. In addition, death was not assessed in this study. Although those findings differed from our network meta-analysis, real-world data from observational studies are still valuable.

Previous meta-analyses on each drug class showed conflicting results of their effects on death [43, 44, 53, 63, 67, 68], in which, the neutral effects of GLP-1 RAs and DPP-4 inhibitors identified in our study were contradicted by their protection in all-cause deaths [63, 67]. We therefore performed a sensitivity analysis to analyse the risk of all-cause mortality by including small trials excluded in our analysis but had been included in these meta-analyses. The overall conclusions did not change, albeit that GLP-1 RAs became significantly better than DPP-4 inhibitors in reducing all-cause mortality (Additional file 1: Table S16 and Online-only reference).

Choosing an antidiabetic drug class with proven benefit in reducing cardiovascular risk and mortality in patients with T2DM is now advocated in guidelines. The newly published CVOTs and renal outcome trials reinforced the confidence in SGLT-2 inhibitors, and to a lesser extent, GLP-1 RAs, as effective treatments. Ongoing trials will provide further evidence on cardiovascular safety and efficacy and potential renal benefits of these drug classes, the validity of the concept of a class effect, and help to refine future guidelines. Head-to-head CVOTs are difficult and expensive to conduct. Therefore, observational studies based on real-world data with sufficient length of follow-up and statistical power compared these new antidiabetic drug classes could be useful to demonstrate benefits at the population level.

Our network meta-analysis including the latest, powerful, and many more CVOTs than previous network meta-analyses has profound and unequivocal clinical implications. They support the new decision pathways of the American College of Cardiology (ACC) [69], which were announced before the release of the results of DECLARE-TIMI 58, Harmony Outcomes, and CARMELINA. Our analysis including all these latest trials is consistent with the ACC report. SGLT-2 inhibitors and GLP-1 RAs should, therefore, be considered evidence-based treatments for T2DM after metformin. SGLT-2 inhibitors can be considered before GLP-1 RAs because of the reduction in mortality. However, whether to choose an SGLT-2 inhibitor or a GLP-1 RA should still be tailored for different patients. The former is oral while the latter is mostly injected. The route of administration may, therefore, influence physician and patient preference. They also have different side effects, such that a patient intolerant of one can be switched to the other. Indeed, the two drug classes can both be given if the glycaemic target is not attained.

## Limitations

Our network meta-analysis has certain limitations. Firstly, there was no head-to-head CVOT directly comparing new antidiabetic drug classes. The comparative effects were generated with indirect evidence. Different drugs were used within each drug class, and there could be within-class differences. Secondly, we did not have patient-level data, limiting the scope for adjustments and subgroup analysis. Finally, there were differences in trial designs, patient characteristics, background therapy, and endpoint definitions.

In conclusion, our network meta-analysis shows that although none of the three new antidiabetic drug classes increases cardiovascular risks, there are clinically significant differences among them. SGLT-2 inhibitors are clearly superior in reducing cardiovascular and all-cause mortality, hospitalisation for HF, and renal events among the new antidiabetic drug classes. Both SGLT-2 inhibitors and GLP-1 RAs can reduce MACE. In terms of cardiovascular and renal outcome, DPP-4 inhibitors are comparable to placebo and are inferior to the other two drug classes. Our findings support SGLT-2 inhibitors as the preferred treatment for patients with T2DM after metformin. Based on the original trial results and meta-analyses including ours, clinical guidelines should advocate the use of antidiabetic drugs that have been proven to reduce cardiovascular events and mortality.

## Supplementary information

**Additional file 1.** Additional Figures and Tables.

## Data Availability

Not applicable.

## Abbreviations

95% CI: 95% confidence intervals
ACC: American College of Cardiology
ADA: American Diabetes Association
CKD: Chronic Kidney Disease
CVOT: Cardiovascular Outcome Trial
DPP-4: dipeptidyl peptidase-4
EASD: European Association for the Study of Diabetes
eGFR: estimated glomerular filtration rate
GLP-1: glucagon-like peptide 1
HF: heart failure
LV: left ventricular
MACE: major adverse cardiovascular events
MI: myocardial infarction
OR: odds ratio
PRISMA: Preferred Reporting Items for Systematic Reviews and Meta-analyses
RA: receptor agonists
SGLT-2: sodium-glucose co-transporter-2
T2DM: type 2 diabetes mellitus
TIMI: Thrombolysis in Myocardial Infarction
