# Supplementary material for: Cardiovascular outcomes in trials of new antidiabetic drug classes: a network meta-analysis
Source: Cardiovasc Diabetol. 2019 Aug 28;18:112. doi: 10.1186/s12933-019-0916-z (PMC6714383; doi:10.1186/s12933-019-0916-z)
Supplement: Supplementary file 1 — Additional file 1. Additional Figures and Tables. [file 12933_2019_916_MOESM1_ESM.docx]

Table of Content

[Table of Content 1](#_Toc16886120)

[Table S1. Definition of the renal composite outcome in each included trial 3](#_Toc16886121)

[Table S2. Summary for blinding, randomisation and placebo control of studies 5](#_Toc16886122)

[Table S3. Risk of Bias Reporting 8](#_Toc16886123)

[Table S4. Patients’ baseline characteristics 9](#_Toc16886124)

[Table S5. Ranking based on simulations 11](#_Toc16886125)

[Table S6. Effect of antidiabetic drugs on frequencies of primary endpoints in patients after excluding the EXAMINE and ELIXA trials established by network meta-analysis using a frequentist approach 13](#_Toc16886126)

[Table S7. Estimates of between trial heterogeneity 15](#_Toc16886127)

[Table S8. Sensitivity analysis of the effect of antidiabetic drugs on frequency of MACE in the comparison between GLP-1 receptor agonist vs. placebo 16](#_Toc16886128)

[Table S9. Sensitivity analysis of the effect of antidiabetic drugs on cardiovascular mortality in the comparison between SGLT-2 inhibitor vs. placebo 17](#_Toc16886129)

[Table S10. Sensitivity analysis of the effect of antidiabetic drugs on all-cause mortality in the comparison between SGLT-2 inhibitor vs. placebo 18](#_Toc16886130)

[Table S11. Sensitivity analysis of the effect of antidiabetic drugs on frequency of hospitalisation for heart failure in the comparison between DPP-4 inhibitor vs. placebo 19](#_Toc16886131)

[Table S12. Effect of antidiabetic drugs on frequencies of primary outcomes in patients established by network meta-analysis using fixed effects model 20](#_Toc16886132)

[Table S13. Effect of antidiabetic drugs on frequencies of primary outcomes in patients established by network meta-analysis using a Bayesian framework with non-informative priors 22](#_Toc16886133)

[Table S14. Analysis of funnel plots in Fig.S2-S8 to assess publication bias 24](#_Toc16886134)

[Table S15. Hazard ratios (antidiabetic drug vs. placebo) of the outcomes evaluated in the cardiovascular outcome trials included 26](#_Toc16886135)

[Table S16. Effect of antidiabetic drugs on frequencies of all-cause mortality in patients established by network meta-analysis after including the non-cardiovascular outcome trials^*^ from previous pairwise and network meta-analyses 28](#_Toc16886136)

[Captions to Additional Figures 29](#_Toc16886137)

[Online-only Reference 30](#_Toc16886138)

[Supplementary Figures 43](#_Toc16886139)

Table S1. Definition of the renal composite outcome in each included trial

| **Study** | **Definition of the renal composite outcome** | |
| --- | --- | --- |
| **GLP-1 RA vs. Placebo** | |  |
| ELIXA | Renal or urinary event | |
| LEADER | Nephropathy, defined as the new onset of macroalbuminuria or a doubling of the serum creatinine level and an eGFR of ≤45 ml per minute per 1.73 m^2^, the need for continuous renal-replacement therapy, or death from renal disease | |
| SUSTAIN-6 | Acute renal failure | |
| HARMONY OUTCOMES | Renal impairment | |
| EXSCEL | 40% eGFR decline, renal replacement, or renal death | |
| **DPP-4 inhibitor vs. Placebo** | |  |
| SAVOR-TIMI | Doubling of creatinine level, initiation of dialysis, renal transplantation, or creatinine >6.0 mg/dl (530 μmol/liter) | |
| EXAMINE | A sustained decrease of eGFR from baseline to less than 60 ml per minute per 1.73 m^2^ of body-surface area | |
| TECOS | Renal failure | |
| CARMELINA | Sustained end-stage renal disease, death due to kidney failure, or sustained decrease of ≥40% in eGFR from baseline | |
| **SGLT-2 inhibitor vs. Placebo** | |  |
| EMPA-REG OUTCOME | A doubling of the serum creatinine level, accompanied by an eGFR of ≤45 ml per minute per 1.73 m^2^ | |
| CANVAS | The renal composite comprising a 40% reduction in eGFR sustained for at least two consecutive measures, the need for renal-replacement therapy (dialysis or transplantation), or death from renal causes (defined as death with a proximate renal cause) | |
| CANVAS-R | The renal composite comprising a 40% reduction in eGFR sustained for at least two consecutive measures, the need for renal-replacement therapy (dialysis or transplantation), or death from renal causes (defined as death with a proximate renal cause) | |
| DECLARE-TIMI 58 | A renal composite outcome, defined as a sustained decrease of 40% or more in estimated glomerular filtration rate (eGFR) to less than 60 ml per minute per 1.73 m^2^ of body-surface area, new end-stage renal disease, or death from renal or cardiovascular causes | |
| CREDENCE | A composite of end-stage kidney disease, doubling of the serum creatinine level, or renal death | |

Abbreviations used in Table S1: DPP-4 = dipeptidyl peptidase-4; eGFR = estimated glomerular filtration rate; GLP-1 = glucagon-like peptide-1; RA = receptor agonist; SGLT-2 = sodium-glucose co-transporter 2.

Table S2. Summary for blinding, randomisation and placebo control of studies

| **Study** | **Blinding** | **Randomisation assignment** | **Presence of Placebo** | **Time of randomisation** | |
| --- | --- | --- | --- | --- | --- |
| **GLP-1 RA vs. Placebo** | | | | |  |
| ELIXA | Double-  blinded | Allocation was conducted through the centralised treatment allocation system | With placebo control | After a placebo run-in period of 7 days (+ 3 days) by a screening visit | |
| LEADER | Double-  blinded | Randomisation was carried out by using an interactive voice response system or web response system with a 1:1 ratio | With placebo control | After an open-label 2-week placebo run-in period | |
| SUSTAIN-6 | Double-  blinded | Randomisation was carried out by using an interactive voice response system or web response system | With placebo control | After Visit 1 screening | |
| HARMONY OUTCOMES | Double-  blinded | Allocation was conducted according to a sequestered, fixed, computer-generated randomisation code that used balanced permuted blocks of treatment group allocations, without stratification | With placebo control | Screening of the potential subjects for study entry and randomisation can occur at the same visit or in close proximity | |
| EXSCEL | Double-  blinded | Not reported | With placebo control | Not reported | |
| **DPP-4 inhibitor vs. Placebo** | | | | |  |
| SAVOR-TIMI 53 | Double-  blinded | Randomisation was conducted by means of a central computerized telephone or web-based system at Visit 1 in blocks of 4 into ratio of 1:1, with stratification according to the qualifying cardiovascular disease state and renal function | With placebo control | After 14 days screening for enrolment | |
| EXAMINE | Double-  blinded | Randomisation was by using an interactive voice response system into a 1:1 ratio stratified based on country and screening renal function | With placebo control | After 14 days screening for enrolment | |
| TECOS | Double-  blinded | Assignments were made by an interactive voice-response system into ratio of 1:1, blocked within each site | With placebo control | At the initial study visit for all patients satisfying all inclusion and exclusion criteria | |
| CARMELINA | Double-  blinded | Randomisation was conducted using an interactive telephone or web-based system into ratio of 1:1, in a block size of 8 | With placebo control | As soon as the visit 1 (safety) laboratory results and eGFR are known and all other eligibility criteria are met | |
| **SGLT-2 inhibitor vs. Placebo** | | | | |  |
| EMPA-REG OUTCOME | Double-  blinded | The blocked randomisation of subjects to the treatment groups was performed via an interactive voice response or interactive web response system | With placebo control | After a 2-week open-label placebo run-in period | |
| CANVAS | Single-blinded | Randomisation was performed centrally through an interactive Web-based response system with the use of a computer-generated randomisation schedule with randomly permuted blocks | With placebo control | Starting from week 13 after enrolment | |
| CANVAS-R | Single-blinded | Randomisation was performed centrally through an interactive Web-based response system with the use of a computer-generated randomisation schedule with randomly permuted blocks | With placebo control | Starting from week 13 after enrolment | |
| DECLARE–TIMI 58 | Double-  blinded | Not reported | With placebo control | After a 4-to-8-week,  single-blind run-in period in the eligible patients to receive placebo, and blood and urine testing | |
| CREDENCE | Double-  blinded | Central randomisation | With placebo control | After a 2-week, single-blind, placebo run-in period | |

Abbreviations used in Table S2: DPP-4 = dipeptidyl peptidase-4; GLP-1 = glucagon-like peptide-1; RA = receptor agonist; SGLT-2 = sodium-glucose co-transporter 2.

Table S3. Risk of Bias Reporting

| **Study** | **Sequence generation** | **Allocation concealment** | **Blinding of participants and personnel** | | **Blinding of outcome** | **Incomplete outcome data** | **Free from other bias** |
| --- | --- | --- | --- | --- | --- | --- | --- |
| **GLP-1 RA vs. Placebo** | | | | | | | |
| ELIXA | Low | Low | Low | Low | | Low | Low |
| LEADER | Low | Low | Low | Low | | Low | Low |
| SUSTAIN-6 | Low | Low | Low | Low | | Low | Low |
| HARMONY OUTCOMES | Low | Low | Low | Low | | Low | Low |
| EXSCEL | Unclear | Low | Low | Low | | Unclear | Unclear |
| **DPP-4 inhibitor vs. Placebo** | | | | | | | |
| SAVOR-TIMI 53 | Low | Low | Low | Low | | Low | Low |
| EXAMINE | Low | Low | Low | Low | | Low | Low |
| TECOS | Low | Low | Low | Low | | Low | Low |
| CARMELINA | Low | Low | Low | Low | | Low | Low |
| **SGLT-2 inhibitor vs. Placebo** | | | | | | | |
| EMPA-REG OUTCOME | Low | Low | Low | Low | | Low | Low |
| CANVAS | Low | Low | Unclear | Low | | Low | Low |
| CANVAS-R | Low | Low | Unclear | Low | | Low | Low |
| DECLARE–TIMI 58 | Unclear | Low | Low | Low | | Low | Low |
| CREDENCE | Low | Low | Low | Low | | Low | Low |

Abbreviations used in Table S3: DPP-4 = dipeptidyl peptidase-4; GLP-1 = glucagon-like peptide-1; RA = receptor agonist; SGLT-2 = sodium-glucose co-transporter 2. Each domain of risk was assigned “Low” for low risk, “Unclear” for unclear risk and “High” for high risk.

Table S4. Patients’ baseline characteristics

| **Study** | **Drug** | **N** | **Age**  **(yrs, mean ± SD)** | **Male (%)** | **Body weight (kg)** | **T2DM duration (yrs)** | **Glycated haemo- globin (%)** | **Current smoker (%)** | **Prior MI (%)** | **Prior HF (%)** | **Prior HTN (%)** | **Prior stroke (%)** | **Prior revascula-risation (%)** | **Prior PCI** | **Follow**  **-up period (yrs)** |
| --- | --- | --- | --- | --- | --- | --- | --- | --- | --- | --- | --- | --- | --- | --- | --- |
| **GLP-1 RA vs. Placebo** | | | | | | | | | | | | | | | |
| ELIXA | Lixisenatide | 3034 | 59.9±9.7 | 69.6 | 84.6±19.2 | 9.2±8.2 | 7.7±1.3 | 11.7 | 22.1 | 22.5 | 75.6 | 4.7 | NR | 67.6 | 2.0 |
|  | Placebo | 3034 | 60.6±9.6 | 69.1 | 85.1±19.6 | 9.4±8.3 | 7.6±1.3 | 11.7 | 22.1 | 22.3 | 77.1 | 6.2 | NR | 66.8 |  |
| LEADER | Liraglutide | 4668 | 64.2 ±7.2 | 64.5 | 91.9±21.2 | 12.8±8.0 | 8.7±1.4 | NR | 31.4 | 17.9 | 5.3 | 15.6 | 39.3 | NR | 3.8 |
|  | Placebo | 4672 | 64.2 ±7.2 | 64.0 | 91.6±20.8 | 12.9±8.1 | 8.7±1.5 | NR | 30.0 | 17.8 | 5.4 | 16.6 | 38.6 | NR |  |
| SUSTAIN-6 | Semaglutide | 1648 | 64.7±7.2 | 61.4 | 92.4±20.7 | 14.2±8.2 | 8.7±1.4 | NR | 32.2 | 23.1 | 93.7 | 14.0 | NR | NR | 2.1 |
|  | Placebo | 1649 | 64.6±7.6 | 60.0 | 91.9±20.6 | 13.6±8.0 | 8.7±1.5 | NR | 32.4 | 24.0 | 91.9 | 15.8 | NR | NR |  |
| HARMONY OUTCOMES | Albiglutide | 4731 | 64.1±8.7 | 70.0 | NR | 14.1±8.6 | 8.76±1.5 | 16.0 | 47.0 | 20.0 | 86.0 | 17.0 | NR | 43.0 | 1.5 |
|  | Placebo | 4732 | 64.2±8.7 | 69.0 | NR | 14.2±8.9 | 8.72±1.5 | 16.0 | 47.0 | 20.0 | 87.0 | 18.0 | NR | 45.0 |  |
| EXSCEL | Exenatide | 5394 | 63 (57-69) | 67.0 | NR | 12 (7-19) | 8.0 (7.3-8.8) | NR | 43.0 | NR | 87.0 | 17.0 | NR | 41.0 | 2.4 |
|  | Placebo | 5388 |  |  |  |  |  |  |  |  |  |  |  |  |  |
| **SGLT-2 inhibitor vs. Placebo** | | | | | | | | | | | | | | | |
| EMPA-REG OUTCOME | Empagliflozin | 4687 | 63.1±8.6 | 71.2 | 86.2±18.9 | NR | 8.1±0.9 | NR | 46.7 | 9.9 | NR | 23.1 | NR | NR | 0.4 |
|  | Placebo | 2333 | 63.2±8.8 | 72.0 | 86.6±19.1 | NR | 8.1±0.9 | NR | 46.4 | 10.5 | NR | 23.7 | NR | NR |  |
| CANVAS | Canagliflozin | 2888 | 62.4±8.0 | 66.1 | NR | 13.4±7.5 | 8.2±0.9 | 17.9 | NR | 11.9 | 87.6 | NR | NR | NR | 1.6 |
|  | Placebo | 1442 | 63.4±8.2 | 63.2 | NR | 13.7±7.8 | 8.2±0.9 | 18.1 | NR | 15.1 | 90.6 | NR | NR | NR |  |
| CANVAS-R | Canagliflozin | 2907 | 64.0±8.2 | 62.8 | NR | 13.7±7.9 | 8.3±1.0 | 17.7 | NR | 16.3 | 91.7 | NR | NR | NR | 1.6 |
|  | Placebo | 2905 | 63.4±8.2 | 63.2 | NR | 13.7±7.8 | 8.2±0.9 | 19.1 | NR | 15.1 | 90.6 | NR | NR | NR |  |
| DECLARE–TIMI 58 | Dapagliflozin | 8582 | 63.9±6.8 | 63.1 | NR | 11.0 (6.0–16.0) | 8.3±1.2 | NR | NR | 9.9 | 7.6 | NR | NR | NR | 4.2 |
|  | Placebo | 8578 | 64.0±6.8 | 62.1 | NR | 10.0 (6.0–16.0) | 8.3±1.2 | NR | NR | 10.2 | 7.6 | NR | NR | NR |  |
| CREDENCE | Canagliflozin | 2202 | 62.9±9.2 | 65.4 | NR | 15.5±8.7 | 8.3±1.3 | 15.5 | 29.7 | 14.9 | 96.8 | 15.5 | NR | NR | 2.62 (median) |
|  | Placebo | 2199 | 63.2±9.2 | 66.7 | NR | 16.0±8.6 | 8.3±1.3 | 13.6 | 30.0 | 14.7 | 96.8 | 16.3 | NR | NR |  |
| **DPP-4 inhibitors vs. Placebo** | | | | | | | | | | | | | | | |
| SAVOR-  TIMI 53 | Saxagliptin | 8280 | 65.1±8.5 | 66.6 | 87.7±18.7 | 10.3 | 8.0±1.4 | NR | 38.0 | 12.8 | 81.2 | NR | 43.1 | NR | 2.1 |
|  | Placebo | 8212 | 65.0±8.6 | 67.3 | 88.1±19.4 | 10.3 | 8.0±1.4 | NR | 37.6 | 12.8 | 82.4 | NR | 43.3 | NR |  |
| EXAMINE | Alogliptin | 2701 | 61.0 | 67.7 | 80.2 | 7.1 | 8.0±1.1 | 13.0 | 88.4 | 28.0 | 82.5 | 7.2 | NR | 62.5 | 3.3 |
|  | Placebo | 2679 | 61.0 | 68.0 | 80.0 | 7.3 | 8.0±1.1 | 14.3 | 87.5 | 27.8 | 83.6 | 7.2 | NR | 62.8 |  |
| TECOS | Sitagliptin | 7332 | 65.4±7.9 | 60.9 | NR | 11.6±8.1 | 7.2±0.5 | 11.8 | 42.7 | 17.8 | NR | NR | NR | 38.9 | 3.0 |
|  | Placebo | 7339 | 65.5±8.0 | 60.5 | NR | 11.6±8.1 | 7.2±0.5 | 11.1 | 42.5 | 18.3 | NR | NR | NR | 40.1 |  |
| CARMELINA | Linagliptin | 3494 | 66.1±9.1 | 61.5 | NR | 15.0±9.6 | 7.9±1.0 | 10.4 | NR | 27.2 | 90.8 | NR | NR | NR | 2.2 (median) |
|  | Placebo | 2485 | 65.6±9.1 | 64.3 | NR | 14.5±9.3 | 8.0±1.0 | 10.0 | NR | 26.4 | 91.2 | NR | NR | NR |  |

Abbreviations used in Table S4: DPP-4 = dipeptidyl peptidase-4; GLP-1 = glucagon-like peptide-1; HF = heart failure; HTN = hypertension; MI = myocardial infarction; NR = not reported; PCI = percutaneous coronary intervention; T2DM = type 2 diabetes mellitus; RA = receptor agonist; SGLT-2 = sodium-glucose co-transporter 2.

Table S5. Ranking based on simulations

|  | **Rank 1 (%)** | **Rank 2 (%)** | **Rank 3 (%)** | **Rank 4 (%)** |
| --- | --- | --- | --- | --- |
| **MACE** |  |  |  |  |
| GLP-1 receptor agonist | 49.50 | 49.00 | 1.40 | 0.10 |
| SGLT-2 inhibitor | 50.20 | 48.25 | 1.50 | 0.05 |
| DPP-4 inhibitor | 0.30 | 2.45 | 50.05 | 47.20 |
| Placebo | 0.00 | 0.30 | 47.05 | 52.65 |
| **Nonfatal myocardial infarction** |  |  |  |  |
| GLP-1 receptor agonist | 35.45 | 58.10 | 5.20 | 1.25 |
| SGLT-2 inhibitor | 63.15 | 30.90 | 4.25 | 1.70 |
| DPP-4 inhibitor | 1.30 | 7.65 | 32.65 | 58.40 |
| Placebo | 0.10 | 3.35 | 57.90 | 38.65 |
| **Nonfatal stroke** |  |  |  |  |
| GLP-1 receptor agonist | 80.55 | 15.35 | 2.90 | 1.20 |
| SGLT-2 inhibitor | 4.70 | 20.85 | 23.50 | 50.95 |
| DPP-4 inhibitor | 14.40 | 40.35 | 21.00 | 24.25 |
| Placebo | 0.35 | 23.45 | 52.60 | 23.60 |
| **Cardiovascular mortality** |  |  |  |  |
| GLP-1 receptor agonist | 20.60 | 63.75 | 12.00 | 3.65 |
| SGLT-2 inhibitor | 77.20 | 20.60 | 1.95 | 0.25 |
| DPP-4 inhibitor | 2.20 | 13.70 | 44.75 | 39.35 |
| Placebo | 0.00 | 1.95 | 41.30 | 56.75 |
| **All-cause mortality** |  |  |  |  |
| GLP-1 receptor agonist | 17.00 | 75.90 | 5.55 | 1.55 |
| SGLT-2 inhibitor | 82.30 | 17.10 | 0.50 | 0.10 |
| DPP-4 inhibitor | 0.70 | 5.30 | 35.50 | 58.50 |
| Placebo | 0.00 | 1.70 | 58.45 | 39.85 |
| **Hospitalisation for heart failure** |  |  |  |  |
| GLP-1 receptor agonist | 0.20 | 85.60 | 9.35 | 4.85 |
| SGLT-2 inhibitor | 99.80 | 0.20 | 0.00 | 0.00 |
| DPP-4 inhibitor | 0.00 | 5.90 | 16.10 | 78.00 |
| Placebo | 0.00 | 8.30 | 74.55 | 17.15 |
| **Renal composite outcome** |  |  |  |  |
| GLP-1 receptor agonist | 0.00 | 96.00 | 3.05 | 0.95 |
| SGLT-2 inhibitor | 100.00 | 0.00 | 0.00 | 0.00 |
| DPP-4 inhibitor | 0.00 | 3.70 | 46.60 | 49.70 |
| Placebo | 0.00 | 0.30 | 50.35 | 49.35 |
| Abbreviations used in Table S5: DPP-4 = dipeptidyl peptidase-4; GLP-1 = glucagon-like peptide-1; MACE = major adverse cardiovascular event (defined as the composite of cardiovascular mortality, nonfatal myocardial infarction and nonfatal stroke); RA = receptor agonist; SGLT-2 = sodium-glucose co-transporter 2.  The numbers in this table represent the probability (in percent) that each antidiabetic drug is best (rank 1), the second highest (rank 2), etc. Rank probabilities sum to 100%, both within a rank over treatments and within a treatment over ranks. | | | | |

Table S6. Effect of antidiabetic drugs on frequencies of primary endpoints in patients after excluding the EXAMINE and ELIXA trials established by network meta-analysis using a frequentist approach

|  | **GLP-1 RA** | **SGLT-2 inhibitor** | **DPP-4 inhibitor** | **Placebo** |
| --- | --- | --- | --- | --- |
| **MACE** | | | | |
| GLP-1 RA | 1.00 | 1.05 (0.95-1.15) | **1.19 (1.08-1.31)** | **1.18 (1.10-1.26)** |
| SGLT-2 inhibitor | 0.96 (0.87-1.05) | 1.00 | **1.13 (1.03-1.25)** | **1.13 (1.05-1.21)** |
| DPP-4 inhibitor | **0.84 (0.76-0.93)** | **0.88 (0.80-0.97)** | 1.00 | 0.99 (0.93-1.07) |
| Placebo | **0.85 (0.79-0.91)** | **0.89 (0.83-0.95)** | 1.01 (0.94-1.08) | 1.00 |
| **Nonfatal myocardial infarction** | | | | |
| GLP-1 RA | 1.00 | 1.06 (0.91-1.29) | 1.16 (0.97-1.38) | **1.16 (1.03-1.30)** |
| SGLT-2 inhibitor | 0.92 (0.77-1.10) | 1.00 | 1.07 (0.88-1.28) | 1.07 (0.94-1.22) |
| DPP-4 inhibitor | 0.86 (0.72-1.03) | 0.94 (0.78-1.13) | 1.00 | 1.00 (0.88-1.14) |
| Placebo | **0.86 (0.77-0.97)** | 0.94 (0.82-1.07) | 1.00 (0.87-1.14) | 1.00 |
| **Nonfatal stroke** | | | | |
| GLP-1 RA | 1.00 | 1.20 (1.00-1.45) | 1.16 (0.95-1.41) | **1.17 (1.03-1.34)** |
| SGLT-2 inhibitor | 0.83 (0.69-1.00) | 1.00 | 0.96 (0.79-1.17) | 0.97 (0.85-1.11) |
| DPP-4 inhibitor | 0.87 (0.71-1.05) | 1.04 (0.86-1.27) | 1.00 | 1.01 (0.88-1.17) |
| Placebo | **0.85 (0.75-0.97)** | 1.03 (0.90-1.17) | 0.99 (0.85-1.14) | 1.00 |
| **Cardiovascular mortality** | | | | |
| GLP-1 RA | 1.00 | 0.94 (0.78-1.12) | 1.17 (0.97-1.41) | 1.14 (1.00-1.31) |
| SGLT-2 inhibitor | 1.07 (0.89-1.28) | 1.00 | **1.24 (1.04-1.48)** | **1.22 (1.08-1.37)** |
| DPP-4 inhibitor | 0.86 (0.71-1.03) | **0.80 (0.67-0.96)** | 1.00 | 0.98 (0.86-1.12) |
| Placebo | 0.88 (0.76-1.00) | **0.82 (0.73-0.93)** | 1.02 (0.90-1.16) | 1.00 |
| **All-cause mortality** | | | | |
| GLP-1 RA | 1.00 | 0.94 (0.83-1.08) | **1.16 (1.02-1.33)** | **1.12 (1.02-1.24)** |
| SGLT-2 inhibitor | 1.06 (0.93-1.21) | 1.00 | **1.23 (1.08-1.41)** | **1.19 (1.09-1.30)** |
| DPP-4 inhibitor | **0.86 (0.75-0.98)** | **0.81 (0.71-0.92)** | 1.00 | 0.97 (0.88-1.06) |
| Placebo | **0.89 (0.81-0.98)** | **0.84 (0.77-0.92)** | 1.04 (0.94-1.14) | 1.00 |
| **Hospitalisation for heart failure** | | | | |
| GLP-1 RA | 1.00 | **0.78 (0.68-0.91)** | **1.22 (1.06-1.40)** | **1.15 (1.06-1.24)** |
| SGLT-2 inhibitor | **1.28 (1.10-1.48)** | 1.00 | **1.55 (1.31-1.84)** | **1.46 (1.29-1.66)** |
| DPP-4 inhibitor | **0.82 (0.72-0.95)** | **0.64 (0.54-0.76)** | 1.00 | 0.94 (0.84-1.06) |
| Placebo | **0.87 (0.80-0.94)** | **0.68 (0.60-0.77)** | 1.06 (0.95-1.19) | 1.00 |
| **Renal composite outcome** | | | | |
| GLP-1 RA | 1.00 | **0.69 (0.59-0.81)** | **1.22 (1.05-1.42)** | **1.17 (1.07-1.29)** |
| SGLT-2 inhibitor | **1.45 (1.24-1.69)** | 1.00 | **1.77 (1.49-2.09)** | **1.70 (1.50-1.91)** |
| DPP-4 inhibitor | **0.82 (0.70-0.95)** | **0.57 (0.48-0.67)** | 1.00 | 0.96 (0.85-1.08) |
| Placebo | **0.85 (0.78-0.94)** | **0.59 (0.52-0.67)** | 1.04 (0.93-1.17) | 1.00 |

Abbreviations used in Table S6: DPP-4 = dipeptidyl peptidase-4; GLP-1 = glucagon-like peptide-1; MACE = major adverse cardiovascular event (defined as the composite of cardiovascular mortality, nonfatal myocardial infarction and nonfatal stroke); RA = receptor agonist; SGLT-2 = sodium-glucose co-transporter 2.

Results are the Odds Ratios (95% Confidence Interval) in the column-defining therapy compared with the Odds Ratios in the row-defining therapy. For efficacy and safety, Odds Ratio <1 favours the column-defining therapy. Significant results are shown in bold.

Table S7. Estimates of between trial heterogeneity

| **Outcomes** | **τ^2^** |
| --- | --- |
| Major adverse cardiovascular events (MACE) | 0.0007 |
| Nonfatal myocardial infarction | 0.0047 |
| Nonfatal stroke | 0 |
| Cardiovascular mortality | 0.0061 |
| All-cause mortality | 0.0019 |
| Hospitalisation for heart failure | 0 |
| Renal composite outcome | 0 |

Table S8. Sensitivity analysis of the effect of antidiabetic drugs on frequency of MACE in the comparison between GLP-1 receptor agonist vs. placebo

| **Study** | **Before excluding study** | | | | | **After excluding study** | | | | |
| --- | --- | --- | --- | --- | --- | --- | --- | --- | --- | --- |
|  | **OR** | **p-value** | **I^2^** | **Chi^2^** | **P-value** | **OR** | **p-value** | **I^2^** | **Chi^2^** | **P-value** |
| ELIXA | 0.87 (0.82-0.93) | <0.0001 | 58% | 9.43 | 0.05 | 0.85 (0.79-0.91) | <0.00001 | 31% | 4.35 | 0.23 |
| LEADER | 0.87 (0.82-0.93) | <0.0001 | 58% | 9.43 | 0.05 | 0.88 (0.82-0.95) | 0.0006 | 68% | 9.29 | 0.03 |
| HARMONY OUTCOME | 0.87 (0.82-0.93) | <0.0001 | 58% | 9.43 | 0.05 | 0.90 (0.84-0.96) | 0.002 | 52% | 6.25 | 0.10 |
| SUSTAIN-6 | 0.87 (0.82-0.93) | <0.0001 | 58% | 9.43 | 0.05 | 0.89 (0.83-0.94) | 0.0002 | 58% | 7.18 | 0.07 |
| EXSCEL | 0.87 (0.82-0.93) | <0.0001 | 58% | 9.43 | 0.05 | 0.86 (0.80-0.93) | <0.0001 | 66% | 8.89 | 0.03 |

Table S9. Sensitivity analysis of the effect of antidiabetic drugs on cardiovascular mortality in the comparison between SGLT-2 inhibitor vs. placebo

| **Study** | **Before excluding study** | | | | | **After excluding study** | | | | |
| --- | --- | --- | --- | --- | --- | --- | --- | --- | --- | --- |
|  | **OR** | **p-value** | **I^2^** | **Chi^2^** | **P-value** | **OR** | **p-value** | **I^2^** | **Chi^2^** | **P-value** |
| EMPA-REG OUTCOME | 0.83 (0.75-0.92) | 0.0006 | 64% | 10.96 | 0.03 | 0.90 (0.80-1.01) | 0.08 | 0% | 2.32 | 0.51 |
| CANVAS | 0.83 (0.75-0.92) | 0.0006 | 64% | 10.96 | 0.03 | 0.82 (0.73-0.92) | 0.0008 | 72% | 10.55 | 0.01 |
| CANVAS-R | 0.83 (0.75-0.92) | 0.0006 | 64% | 10.96 | 0.03 | 0.83 (0.74-0.93) | 0.0008 | 72% | 10.90 | 0.01 |
| DECLARE-TIMI 58 | 0.83 (0.75-0.92) | 0.0006 | 64% | 10.96 | 0.03 | 0.76 (0.67-0.87) | <0.0001 | 48% | 5.78 | 0.12 |
| CREDENCE | 0.83 (0.75-0.92) | 0.0006 | 64% | 10.96 | 0.03 | 0.85 (0.75-0.95) | 0.004 | 72% | 10.61 | 0.01 |

Table S10. Sensitivity analysis of the effect of antidiabetic drugs on all-cause mortality in the comparison between SGLT-2 inhibitor vs. placebo

| **Study** | **Before excluding study** | | | | | **After excluding study** | | | | |
| --- | --- | --- | --- | --- | --- | --- | --- | --- | --- | --- |
|  | OR | p-value | I^2^ | Chi^2^ | P-value | OR | p-value | I^2^ | Chi^2^ | P-value |
| EMPA-REG OUTCOME | 0.85 (0.78-0.92) | <0.0001 | 50% | 8.07 | 0.09 | 0.89 (0.81-0.97) | 0.009 | 0% | 1.28 | 0.73 |
| CANVAS | 0.85 (0.78-0.92) | <0.0001 | 50% | 8.07 | 0.09 | 0.85 (0.78-0.93) | 0.0003 | 63% | 8.06 | 0.04 |
| CANVAS-R | 0.85 (0.78-0.92) | <0.0001 | 50% | 8.07 | 0.09 | 0.84 (0.77-0.91) | <0.0001 | 60% | 7.57 | 0.06 |
| DECLARE-TIMI 58 | 0.85 (0.78-0.92) | <0.0001 | 50% | 8.07 | 0.09 | 0.79 (0.71-0.88) | <0.0001 | 35% | 4.63 | 0.20 |
| CREDENCE | 0.85 (0.78-0.92) | <0.0001 | 50% | 8.07 | 0.09 | 0.85 (0.78-0.93) | 0.0003 | 62% | 7.98 | 0.05 |

Table S11. Sensitivity analysis of the effect of antidiabetic drugs on frequency of hospitalisation for heart failure in the comparison between DPP-4 inhibitor vs. placebo

| **Study** | **Before excluding study** | | | | | **After excluding study** | | | | |
| --- | --- | --- | --- | --- | --- | --- | --- | --- | --- | --- |
|  | **OR** | **p-value** | **I^2^** | **Chi^2^** | **P-value** | **OR** | **p-value** | **I^2^** | **Chi^2^** | **P-value** |
| EXAMINE | 1.06 (0.96-1.18) | 0.24 | 54% | 6.45 | 0.09 | 1.06 (0.95-1.18) | 0.27 | 69% | 6.45 | 0.04 |
| SAVOR-TIMI 53 | 1.06 (0.96-1.18) | 0.24 | 54% | 6.45 | 0.09 | 0.97 (0.86-1.10) | 0.68 | 0% | 0.77 | 0.68 |
| TECOS | 1.06 (0.96-1.18) | 0.24 | 54% | 6.45 | 0.09 | 1.09 (0.97-1.23) | 0.15 | 66% | 5.82 | 0.05 |
| CARMELINA | 1.06 (0.96-1.18) | 0.24 | 54% | 6.45 | 0.09 | 1.12 (1.00-1.26) | 0.06 | 42% | 3.45 | 0.18 |

Table S12. Effect of antidiabetic drugs on frequencies of primary outcomes in patients established by network meta-analysis using fixed effects model

|  | **GLP-1 RA** | **SGLT-2 inhibitor** | **DPP-4 inhibitor** | **Placebo** |
| --- | --- | --- | --- | --- |
| **MACE** | | | | |
| GLP-1 RA | 1.00 | 1.01 (0.92-1.11) | **1.14 (1.04-1.25)** | **1.14 (1.07-1.22)** |
| SGLT-2 inhibitor | 0.99 (0.90-1.08) | 1.00 | **1.12 (1.02-1.24)** | **1.13 (1.06-1.21)** |
| DPP-4 inhibitor | **0.88 (0.80-0.96)** | **0.89 (0.81-0.98)** | 1.00 | 1.00 (0.94-1.07) |
| Placebo | **0.87 (0.82-0.93)** | **0.89 (0.83-0.95)** | 1.00 (0.94-1.06) | 1.00 |
| **Nonfatal myocardial infarction** | | | | |
| GLP-1 RA | 1.00 | 1.03 (0.90-1.17) | 1.11 (0.98-1.26) | **1.10 (1.02-1.20)** |
| SGLT-2 inhibitor | 0.97 (0.85-1.11) | 1.00 | 1.08 (0.94-1.25) | 1.07 (0.97-1.19) |
| DPP-4 inhibitor | 0.90 (0.79-1.02) | 0.92 (0.80-1.06) | 1.00 | 0.99 (0.90-1.09) |
| Placebo | **0.91 (0.84-0.98)** | 0.93 (0.84-1.03) | 1.01 (0.92-1.11) | 1.00 |
| **Nonfatal stroke** | | | | |
| GLP-1 RA | 1.00 | 1.17 (0.98-1.41) | 1.12 (0.93-1.35) | **1.14 (1.01-1.29)** |
| SGLT-2 inhibitor | 0.85 (0.71-1.02) | 1.00 | 0.95 (0.79-1.16) | 0.97 (0.85-1.11) |
| DPP-4 inhibitor | 0.89 (0.74-1.08) | 1.05 (0.87-1.27) | 1.00 | 1.02 (0.89-1.18) |
| Placebo | **0.88 (0.77-0.99)** | 1.03 (0.90-1.17) | 0.98 (0.85-1.13) | 1.00 |
| **Cardiovascular mortality** | | | | |
| GLP-1 RA | 1.00 | 0.94 (0.81-1.08) | 1.13 (0.98-1.30) | **1.13 (1.02-1.26)** |
| SGLT-2 inhibitor | 1.07 (0.93-1.23) | 1.00 | **1.20 (1.05-1.38)** | **1.21 (1.10-1.33)** |
| DPP-4 inhibitor | 0.89 (0.77-1.02) | **0.83 (0.73-0.95)** | 1.00 | 1.01 (0.92-1.10) |
| Placebo | **0.88 (0.80-0.98)** | **0.83 (0.75-0.91)** | 0.99 (0.91-1.08) | 1.00 |
| **All-cause mortality** | | | | |
| GLP-1 RA | 1.00 | 0.95 (0.85-1.06) | **1.14 (1.02-1.27)** | **1.12 (1.03-1.21)** |
| SGLT-2 inhibitor | 1.06 (0.94-1.18) | 1.00 | **1.20 (1.08-1.34)** | **1.18 (1.09-1.28)** |
| DPP-4 inhibitor | **0.88 (0.79-0.98)** | **0.83 (0.75-0.93)** | 1.00 | 0.98 (0.91-1.06) |
| Placebo | **0.89 (0.83-0.97)** | **0.85 (0.78-0.92)** | 1.02 (0.94-1.09) | 1.00 |
| **Hospitalisation for heart failure** | | | | |
| GLP-1 RA | 1.00 | **0.79 (0.69-0.90)** | **1.22 (1.08-1.37)** | **1.15 (1.08-1.22)** |
| SGLT-2 inhibitor | **1.27 (1.11-1.45)** | 1.00 | **1.55 (1.33-1.81)** | **1.46 (1.30-1.64)** |
| DPP-4 inhibitor | **0.82 (0.73-0.92)** | **0.64 (0.55-0.75)** | 1.00 | 0.94 (0.85-1.04) |
| Placebo | **0.87 (0.82-0.93)** | **0.68 (0.61-0.77)** | 1.06 (0.96-1.18) | 1.00 |
| **Renal composite outcome** | | | | |
| GLP-1 RA | 1.00 | **0.69 (0.59-0.80)** | **1.16 (1.03-1.31)** | **1.16 (1.06-1.27)** |
| SGLT-2 inhibitor | **1.46 (1.25-1.70)** | 1.00 | **1.69 (1.48-1.96)** | **1.70 (1.50-1.91)** |
| DPP-4 inhibitor | **0.86 (0.76-0.98)** | **0.59 (0.51-0.69)** | 1.00 | 1.00 (0.92-1.09) |
| Placebo | **0.86 (0.78-0.94)** | **0.59 (0.52-0.67)** | 1.00 (0.92-1.08) | 1.00 |

Abbreviations used in Table S12: DPP-4 = dipeptidyl peptidase-4; GLP-1 = glucagon-like peptide-1; MACE = major adverse cardiovascular event (defined as the composite of cardiovascular mortality, nonfatal myocardial infarction and nonfatal stroke); RA = receptor agonist; SGLT-2 = sodium-glucose co-transporter 2.

Results are the Odds Ratios (95% Confidence Interval) in the column-defining therapy compared with the Odds Ratios in the row-defining therapy. For efficacy and safety, Odds Ratio <1 favours the column-defining therapy. Significant results are shown in bold.

Table S13. Effect of antidiabetic drugs on frequencies of primary outcomes in patients established by network meta-analysis using a Bayesian framework with non-informative priors

|  | **GLP-1 RA** | **SGLT-2 inhibitor** | **DPP-4 inhibitor** | **Placebo** |
| --- | --- | --- | --- | --- |
| **MACE** | | | | |
| GLP-1 RA | 1.00 | 1.00 (0.88-1.10) | 1.10 (1.00-1.30) | **1.10 (1.10-1.30)** |
| SGLT-2 inhibitor | 1.00 (0.88-1.10) | 1.00 | **1.10 (1.00-1.30)** | **1.10 (1.10-1.30)** |
| DPP-4 inhibitor | **0.87 (0.77-0.99)** | **0.88 (0.76-0.99)** | 1.00 | 1.00 (0.91-1.10) |
| Placebo | **0.87 (0.79-0.95)** | **0.87 (0.79-0.95)** | 1.00 (0.91-1.10) | 1.00 |
| **Nonfatal myocardial infarction** | | | | |
| GLP-1 RA | 1.00 | 0.97 (0.82-1.20) | 1.10 (0.96-1.30) | 1.10 (1.00-1.20) |
| SGLT-2 inhibitor | 1.00 (0.87-1.20) | 1.00 | 1.20 (0.98-1.40) | 1.10 (1.00-1.30) |
| DPP-4 inhibitor | 0.89 (0.75-1.00) | 0.86 (0.71-1.00) | 1.00 | 0.98 (0.87-1.10) |
| Placebo | 0.90 (0.80-1.00) | 0.87 (0.76-1.00) | 1.00 (0.90-1.20) | 1.00 |
| **Nonfatal stroke** | | | | |
| GLP-1 RA | 1.00 | 1.20 (0.94-1.50) | 1.10 (0.88-1.40) | 1.10 (0.98-1.30) |
| SGLT-2 inhibitor | 0.85 (0.69-1.10) | 1.00 | 0.94 (0.75-1.20) | 0.97 (0.83-1.10) |
| DPP-4 inhibitor | 0.90 (0.77-1.10) | 1.10 (0.83-1.30) | 1.00 | 1.00 (0.87-1.20) |
| Placebo | 0.88 (0.75-1.00) | 1.00 (0.87-1.20) | 0.97 (0.82-1.20) | 1.00 |
| **Cardiovascular mortality** | | | | |
| GLP-1 RA | 1.00 | 0.93 (0.76-1.10) | 1.10 (0.90-1.40) | 1.10 (0.97-1.30) |
| SGLT-2 inhibitor | 1.10 (0.89-1.30) | 1.00 | 1.20 (0.98-1.50) | **1.20 (1.10-1.40)** |
| DPP-4 inhibitor | 0.90 (0.74-1.10) | 0.84 (0.69-1.00) | 1.00 | 1.00 (0.88-1.20) |
| Placebo | 0.88 (0.77-1.00) | **0.82 (0.71-0.94)** | 0.98 (0.84-1.10) | 1.00 |
| **All-cause mortality** | | | | |
| GLP-1 RA | 1.00 | 0.93 (0.79-1.10) | 1.10 (0.95-1.30) | 1.10 (0.98-1.20) |
| SGLT-2 inhibitor | 1.10 (0.93-1.30) | 1.00 | **1.20 (1.00-1.40)** | **1.20 (1.10-1.30)** |
| DPP-4 inhibitor | 0.89 (0.76-1.10) | **0.83 (0.71-0.97)** | 1.00 | 0.99 (0.89-1.10) |
| Placebo | 0.90 (0.81-1.10) | **0.84 (0.75-0.93)** | 1.00 (0.90-1.10) | 1.00 |
| **Hospitalisation for heart failure** | | | | |
| GLP-1 RA | 1.00 | **0.74 (0.60-0.89)** | 1.10 (0.94-1.40) | 1.10 (0.94-1.20) |
| SGLT-2 inhibitor | **1.40 (1.10-1.70)** | 1.00 | **1.60 (1.30-1.90)** | **1.50 (1.30-1.70)** |
| DPP-4 inhibitor | 0.88 (0.72-1.10) | **0.64 (0.53-0.79)** | 1.00 | 0.94 (0.83-1.10) |
| Placebo | 0.92 (0.81-1.10) | **0.68 (0.59-0.79)** | 1.10 (0.93-1.20) | 1.00 |
| **Renal composite outcome** | | | | |
| GLP-1 RA | 1.00 | **0.69 (0.56-0.82)** | 1.20 (0.98-1.40) | **1.20 (1.00-1.30)** |
| SGLT-2 inhibitor | **1.50 (1.20-1.80)** | 1.00 | **1.70 (1.40-2.00)** | **1.70 (1.50-1.90)** |
| DPP-4 inhibitor | 0.86 (0.73-1.00) | **0.59 (0.49-0.71)** | 1.00 | 1.00 (0.89-1.10) |
| Placebo | **0.86 (0.77-0.97)** | **0.59 (0.51-0.68)** | 1.00 (0.89-1.10) | 1.00 |

Abbreviations used in Table S13: DPP-4 = dipeptidyl peptidase-4; GLP-1 = glucagon-like peptide-1; MACE = major adverse cardiovascular events (defined as the composite of cardiovascular mortality, nonfatal myocardial infarction and nonfatal stroke); RA = receptor agonist; SGLT-2 = sodium-glucose co-transporter 2.

Data are Odds Ratio (95% Confidence Interval). All the drugs were compared with placebo.

**Table S14. Analysis of funnel plots in Fig.S2-S8 to assess publication bias**

| Suppl.  Figure | Begg’s rank correlation test | | Egger’s regression intercept | Trim and fill | | |
| --- | --- | --- | --- | --- | --- | --- |
|  |  |  |  | Before trimming | After trimming | |
|  | τ without continuity correction | p-value | p-value | Observed Point estimate (LL-UL) | Trimming direction | Adjusted Point estimate (LL-UL) |
| Fig.S2A | 0.200 | 0.624 | 0.437 | 0.881 (0.796-0.976) | Left | 0.968 (0.877-0.794) |
| Fig.S2B | -1.000 | 0.415 | 0.516 | 0.990 (0.891-1.099) | NA | 0.990 (0.891-1.099) |
| Fig.S2C | -0.200 | 0.624 | 0.599 | 0.817 (0.683-0.977) | Left | 0.728 (0.596-0.888) |
| Fig.S3A | 0.800 | 0.050 | 0.059 | 0.894 (0.827-0.967) | Left | 0.876 (0.815-0.940) |
| Fig.S3B | -0.667 | 0.174 | 0.271 | 1.013 (0.934-1.100) | Right | 1.035 (0.946-1.133) |
| Fig.S3C | 0.000 | 1.000 | 0.530 | 0.851 (0.776-0.934) | NA | 0.851 (0.776-0.934) |
| Fig.S4A | -0.600 | 0.142 | 0.100 | 0.903 (0.832-0.981) | Right | 0.913 (0.842-0.989) |
| Fig.S4B | 0.667 | 0.174 | 0.009 | 1.008 (0.916-1.110) | Left | 0.982 (0.890-1.084) |
| Fig.S4C | -0.667 | 0.308 | 0.071 | 0.879 (0.792-0.975) | Right | 0.884 (0.806-0.968) |
| Fig.S5A | 0.000 | 1.000 | 0.738 | 0.889 (0.782-1.012) | NA | 0.889 (0.782-1.012) |
| Fig.S5B | 0.000 | 1.000 | 0.526 | 0.979 (0.851-1.125) | Right | 1.010 (0.891-1.145) |
| Fig.S5C | -0.667 | 0.174 | 0.837 | 1.027 (0.897-1.176) | Right | 1.067 (0.920-1.239) |
| Fig.S6A | -0.400 | 0.327 | 0.483 | 0.884 (0.813-0.962) | Right | 0.902 (0.824-0.987) |
| Fig.S6B | 0.000 | 1.000 | 0.706 | 0.998 (0.936-1.064) | Right | 1.006 (0.948-1.068) |
| Fig.S6C | -0.600 | 0.142 | 0.141 | 0.827 (0.733-0.933) | NA | 0.827 (0.733-0.933) |
| Fig.S7A | 0.800 | 0.050 | 0.069 | 0.937 (0.848-1.035) | Left | 0.913 (0.837-0.996) |
| Fig.S7B | -0.333 | 0.497 | 0.844 | 1.057 (0.907-1.232) | NA | 1.057 (0.907-1.232) |
| Fig.S7C | -0.600 | 0.142 | 0.410 | 0.685 (0.609-0.770) | Right | 0.698 (0.623-0.781) |
| Fig.S8A | 0.000 | 1.000 | 0.722 | 0.590 (0.522-0.666) | NA | 0.590 (0.522-0.666) |
| Fig.S8B | 0.000 | 1.000 | 0.820 | 0.997 (0.918-1.082) | NA | 0.997 (0.918-1.082) |
| Fig.S8C | -0.200 | 0.624 | 0.794 | 0.633 (0.552-0.727) | NA | 0.633 (0.552-0.727) |

Abbreviations used in Table S14: LL = Lower limit; NA= Not Applicable; Suppl. = Supplementary; UL = Upper limit.

Table S15. Hazard ratios (antidiabetic drug vs. placebo) of the outcomes evaluated in the cardiovascular outcome trials included

| **Studies** | **HR (95% CI) of nonfatal MI** | **HR (95% CI) of nonfatal stroke** | **HR (95% CI) of hospitalisation for HF** | **HR (95% CI) of composite renal composite outcome^†^** | **HR (95% CI) of cardiovascular mortality** |
| --- | --- | --- | --- | --- | --- |
| **GLP-1 RA vs. Placebo** | | | | | |
| ELIXA | 1.03 (0.87-1.22) | 1.12 (0.79-1.58) | 0.96 (0.75-1.23) | Not applicable^‡^ | 0.98 (0.78-1.22) |
| LEADER | 0.88 (0.75-1.03) | 0.89 (0.72-1.11) | 0.87 (0.73-1.05) | 0.78 (0.67-0.92) | 0.78 (0.66-0.93) |
| SUSTAIN-6 | 0.74 (0.51-1.08) | 0.61 (0.38-0.99) | 1.11 (0.77-1.61) | 0.64 (0.46-0.88) | 0.98 (0.65-1.48) |
| Harmony Outcomes | 0.75 (0.61-0.90) | 0.86 (0.66-1.14) | 0.85 (0.70-1.04) | 0.87 (0.75-1.02)^§^ | 0.93 (0.73-1.19) |
| EXSCEL | 0.95 (0.83-1.09) | 0.82 (0.67-1.02) | 0.95 (0.78-1.16) | 0.88 (0.74-1.05) | 0.89 (0.76-1.04) |
| **DPP-4 inhibitors vs. Placebo** | | | | | |
| SAVOR-TIMI 53 | 0.95 (0.80-1.12) | 1.11 (0.88-1.39) | 1.27 (1.07-1.51) | 1.08 (0.88-1.32) | 1.03 (0.87-1.22) |
| EXAMINE | 1.10 (0.88-1.37) | 0.97 (0.58-1.62) | 1.07 (0.79-1.46) | Not applicable | 0.72 (0.52-1.00) |
| TECOS | 0.96 (0.81-1.13) | 0.93 (0.75-1.16) | 1.00 (0.83-1.20) | Not applicable | 1.04 (0.87-1.24) |
| CARMELINA | 1.15 (0.91-1.45) | 0.88 (0.63-1.23) | 0.90 (0.74-1.08) | 0.98 (0.82-1.18) | 0.96 (0.81-1.14) |
| **SGLT-2 inhibitor vs. Placebo** | | | | | |
| EMPA-REG OUTCOME | 0.87 (0.70-1.09) | 1.24 (0.92-1.67) | 0.65 (0.50-0.85) | 0.54 (0.70-0.75) | 0.62 (0.49-0.77) |
| CANVAS | 0.85 (0.61-1.19) | 0.97 (0.70-1.35) | 0.56 (0.38-0.83) | 0.56 (0.41-0.75) | 0.88 (0.70-1.10) |
| CANVAS-R | 0.85 (0.69-1.05) | 0.82 (0.57-1.18) | 0.67 (0.52-0.87) | 0.71 (0.45-1.11) | 0.86 (0.61-1.22) |
| DECLARE–TIMI 58 | 0.89 (0.77-1.01) | 1.01 (0.84-1.21) | 0.73 (0.61-0.88) | 0.53 (0.43-0.66) | 0.98 (0.82-1.17) |
| CREDENCE | Not applicable | Not applicable | 0.61 (0.47-0.80) | 0.66 (0.53-0.81) | 0.78 (0.61-1.00) |

Abbreviations used in Table S15: DPP-4 = dipeptidyl peptidase-4; GLP-1 = glucagon-like peptide-1; HF = heart failure; HR = hazard ratio; MI = myocardial infarction; RA = receptor agonist; SGLT-2 = sodium-glucose co-transporter 2; 95% CI = 95% confidence interval.

^†^ Renal composite outcome was defined as a composite of adjudication-confirmed end-stage renal disease, death due to renal failure, or a sustained decrease of at least 40% in estimated glomerular filtration rate from baseline to less than 60 ml per minute per 1.73 m^2^ of body-surface area.

^‡^ Except for the EXSCEL trial, the other trials reported the frequencies of the composite adverse outcome instead of the HR.

^§^ Risk ratio was reported.

Table S16. Effect of antidiabetic drugs on frequencies of all-cause mortality in patients established by network meta-analysis after including the non-cardiovascular outcome trials^*^ from previous pairwise and network meta-analyses

|  | **GLP-1 RA** | **SGLT-2 inhibitor** | **DPP-4 inhibitor** | **Placebo** |
| --- | --- | --- | --- | --- |
| **GLP-1 RA** | 1.00 | 0.96 (0.86-1.07) | **1.15 (1.04-1.28)** | **1.13 (1.05-1.22)** |
| **SGLT-2 inhibitor** | 1.04 (0.93-1.16) | 1.00 | **1.20 (1.08-1.33)** | **1.18 (1.09-1.27)** |
| **DPP-4 inhibitor** | **0.87 (0.78-0.96)** | **0.83 (0.75-0.93)** | 1.00 | 0.98 (0.91-1.05) |
| **Placebo** | **0.88 (0.82-0.96)** | **0.85 (0.79-0.92)** | 1.02 (0.95-1.10) | 1.00 |

Abbreviations used in Table S16: DPP-4 = dipeptidyl peptidase-4; GLP-1 = glucagon-like peptide-1; RA = receptor agonist; SGLT-2 = sodium-glucose co-transporter 2.

Results are the Odds Ratios (95% Confidence Interval) in the column-defining therapy compared with the Odds Ratios in the row-defining therapy. For efficacy and safety, Odds Ratio <1 favours the column-defining therapy. Significant results are shown in bold.

^*^ The included trials are listed in the Online-only Reference 1-146.

Captions to Supplementary Figures

Fig.S1. Flow diagram of scientific literature search and study selection

Fig.S2. Funnel plot showing publication bias for the effect of antidiabetic drugs on cardiovascular mortality rate in type 2 diabetes mellitus patients

(A) GLP-1 receptor agonists; (B) DPP-4 inhibitors; (C) SGLT-2 inhibitors.

Fig.S3. Funnel plot showing publication bias for the effect of antidiabetic drugs on all-cause mortality rate in type 2 diabetes mellitus patients

(A) GLP-1 receptor agonists; (B) DPP-4 inhibitors; (C) SGLT-2 inhibitors.

Fig.S4. Funnel plot showing publication bias for the effect of antidiabetic drugs on frequencies of nonfatal myocardial infarction in type 2 diabetes mellitus patients

(A) GLP-1 receptor agonists; (B) DPP-4 inhibitors; (C) SGLT-2 inhibitors.

Fig.S5. Funnel plot showing publication bias for the effect of antidiabetic drugs on frequencies of nonfatal stroke in type 2 diabetes mellitus patients

(A) GLP-1 receptor agonists; (B) DPP-4 inhibitors; (C) SGLT-2 inhibitors.

Fig.S6. Funnel plot showing publication bias for the effect of antidiabetic drugs on frequencies of MACE in type 2 diabetes mellitus patients

(A) GLP-1 receptor agonists; (B) DPP-4 inhibitors; (C) SGLT-2 inhibitors.

MACE = major adverse cardiovascular events (defined as the composite of cardiovascular mortality, nonfatal myocardial infarction and nonfatal stroke).

Fig.S7. Funnel plot showing publication bias for the effect of antidiabetic drugs on frequencies of hospitalisation for heart failure in type 2 diabetes mellitus patients

(A) GLP-1 receptor agonists; (B) DPP-4 inhibitors; (C) SGLT-2 inhibitors.

Fig.S8. Funnel plot showing publication bias for the effect of antidiabetic drugs on frequencies of renal composite outcome in type 2 diabetes mellitus patients

(A) GLP-1 receptor agonists; (B) DPP-4 inhibitors; (C) SGLT-2 inhibitors.

Online-only Reference

1 Barnett AH, Patel S, Harper R, et al. Linagliptin monotherapy in type 2 diabetes patients for whom metformin is inappropriate: an 18-week randomized, double-blind, placebo-controlled phase III trial with a 34-week active-controlled extension. *Diabetes Obese Metab* 2012; 14: 1145-1154.

2 Bosi E, Dotta F, Jia Y, et al. Vildagliptin plus metformin combination therapy provides superior glycaemic control to individual monotherapy in treatment-naive patients with type 2 diabetes mellitus. *Diabetes Obes Metab* 2009; 11: 506-515.

3 DeFronzo RA, Burant CF, Fleck P, et al. Efficacy and tolerability of the DPP-4 inhibitor alogliptin combined with pioglitazone, in metformin-treated patients with type 2 diabetes. *J Clin Endocrinol Metab* 2012; 97: 1615-1622.

4 Fonseca V, Schweizer A, Albrecht D, et al. Addition of vildagliptin to insulin improves glycaemic control in type 2 diabetes. *Diabetologia* 2007; 50: 1148-1155.

5 Frederich R, McNeill R, Berglind N, et al. The efficacy and safety of the dipeptidyl peptidase-4 inhibitor saxagliptin in treatment-naïve patients with type 2 diabetes mellitus: a randomized controlled trial. *Diabetol Metab Syndr* 2012; 4: 36.

6 Rosenstock J, Gross JL, Aguilar-Salinas C, et al. Long-term 4-year safety of saxagliptin in drug-naive and metformin-treated patients with Type 2 diabetes. *Diabet Med* 2013; 30: 1472-1476.

7 Scherbaum WA, Schweizer A, Mari A, et al. Efficacy and tolerability of vildagliptin in drug-naïve patients with type 2 diabetes and mild hyperglycaemia. *Diabetes Obes Metab* 2008; 10: 675-682.

8 Pan CY, Yang W, Tou C, et al. Efficacy and safety of saxagliptin in drug-naïve Asian patients with type 2 diabetes mellitus: a randomized controlled trial. *Diabetes Metab Res Rev* 2012; 28: 268-275.

9 Cefalu WT, Leiter LA, de Bruin TW, et al. Dapagliflozin's Effects on Glycemia and Cardiovascular Risk Factors in High-Risk Patients With Type 2 Diabetes: A 24-Week, Multicenter, Randomized, Double-Blind, Placebo-Controlled Study With a 28-Week Extension. *Diabetes Care* 2015; 38: 1218-1227.

10 Jabbour SA, Hardy E, Sugg J, Parikh S. Dapagliflozin is effective as add-on therapy to sitagliptin with or withoutmetformin: A 24-Week, multicenter, randomized, double-blind, placebo-controlled study. *Diabetes Care* 2014; 37: 740-750.

11 Häring HU, Merker L, Seewaldt-Becker E, et al. Empagliflozin as add-on to metformin plus sulfonylurea in patients with type 2 diabetes: A 24-week, randomized, double-blind, placebo-controlled trial. *Diabetes Care* 2013; 36: 3396-3404.

12 Kovacs CS, Seshiah V, Swallow R, et al. Empagliflozin improves glycaemic and weight control as add-on therapy to pioglitazone or pioglitazone plus metformin in patients with type 2 diabetes: A 24-week, randomized, placebo-controlled trial. *Diabetes Obes Metab* 2014; 16: 147-158.

13 Rosenstock J, Jelaska A, Frappin G, et al. Improved glucose control with weight loss, lower insulin doses, and no increased hypoglycemia with empagliflozin added to titrated multiple daily injections of insulin in obese inadequately controlled type 2 diabetes. *Diabetes Care* 2014; 37: 1815-1823.

14 Nauck M, Frid A, Hermansen K, et al. Efficacy and safety comparison of liraglutide, glimepiride, and placebo, all in combination with metformin, in type 2 diabetes: the LEAD (liraglutide effect and action in diabetes)-2 study. *Diabetes Care* 2009; 32(1): 84–90.

15 ClinicalTrials.gov Identifier: NCT01098539. <https://clinicaltrials.gov/ct2/show/NCT01098539> (accessed November 20, 2018).

16 Pinget M, Goldenberg R, Niemoeller E, Muehlen-Bartmer I, Guo H, Aronson R. Efficacy and safety of lixisenatide once daily versus placebo in type 2 diabetes insufficiently controlled on pioglitazone (GetGoal-P). *Diabetes Obes Metab* 2013; 15(11): 1000-7.

17 Pratley RE, Fleck P, Wilson C. Efficacy and safety of initial combination therapy with alogliptin plus metformin versus either as monotherapy in drug-naive patients with type 2 diabetes: a randomized, double-blind, 6-month study. *Diabetes Obes Metab* 2014; 16(7): 613-621.

18 Kaku K, Itayasu T, Hiroi S, Hirayama M, Seino Y. Efficacy and safety of alogliptin added to pioglitazone in Japanese patients with type 2 diabetes: a randomized, double-blind, placebo-controlled trial with an open-label long-term extension study. *Diabetes Obes Metab* 2011; 13(11): 1028-1035.

19 Nauck MA, Ellis GC, Fleck PR, Wilson CA, Mekki Q. Efficacy and safety of adding the dipeptidyl peptidase-4 inhibitor alogliptin to metformin therapy in patients with type 2 diabetes inadequately controlled with metformin monotherapy: a multicentre, randomised, double-blind, placebo-controlled study. *Int J Clin Pract* 2009; 63(1): 46-55.

20 Pratley RE, Reusch JE, Fleck PR, Wilson CA, Mekki Q. Efficacy and safety of the dipeptidyl peptidase-4 inhibitor alogliptin added to pioglitazone in patients with type 2 diabetes: a randomized, double-blind, placebo-controlled study. *Curr Med Res Opin* 2009; 25(10): 2361-2371.

21 Rosenstock J, Rendell MS, Gross JL, Fleck PR, Wilson CA, Mekki Q. Alogliptin added to insulin therapy in patients with type 2 diabetes reduces HbA(1C) without causing weight gain or increased hypoglycaemia. *Diabetes Obes Metab* 2009; 11(12): 1145-1152.

22 Bajaj M, Gilman R, Patel S, Kempthorne-Rawson J, Lewis-D'Agostino D, Woerle HJ. Linagliptin improved glycaemic control without weight gain or hypoglycaemia in patients with type 2 diabetes inadequately controlled by a combination of metformin and pioglitazone: a 24-week randomized, double-blind study. *Diabet Med* 2014; 31(12): 1505-1514.

23 Barnett AH, Huisman H, Jones R, von Eynatten M, Patel S, Woerle HJ. Linagliptin for patients aged 70 years or older with type 2 diabetes inadequately controlled with common antidiabetes treatments: a randomised, double-blind, placebo-controlled trial. *Lancet* 2013; 382(9902): 1413-1423.

24 McGill JB, Sloan L, Newman J, et al. Long-term efficacy and safety of linagliptin in patients with type 2 diabetes and severe renal impairment: a 1-year, randomized, double-blind, placebo-controlled study. Diabetes Care 2013; 36(2): 237-244.

25 Yki-Jarvinen H, Rosenstock J, Duran-Garcia S, et al. Effects of adding linagliptin to basal insulin regimen for inadequately controlled type 2 diabetes: a >/=52-week randomized, double-blind study. *Diabetes Care* 2013; 36(12): 3875-3881.

26 Gallwitz B, Rosenstock J, Rauch T, et al. 2-year efficacy and safety of linagliptin compared with glimepiride in patients with type 2 diabetes inadequately controlled on metformin: a randomised, double-blind, non-inferiority trial. *Lancet* 2012; 380(9840): 475-483.

27 Gomis R, Espadero RM, Jones R, Woerle HJ, Dugi KA. Efficacy and safety of initial combination therapy with linagliptin and pioglitazone in patients with inadequately controlled type 2 diabetes: a randomized, double-blind, placebo-controlled study. *Diabetes Obes Metab* 2011; 13(7): 653-661.

28 Haak T, Meinicke T, Jones R, Weber S, von Eynatten M, Woerle HJ. Initial combination of linagliptin and metformin improves glycaemic control in type 2 diabetes: a randomized, double-blind, placebo-controlled study. *Diabetes Obes Metab* 2012; 14(6): 565-574.

29 DeFronzo RA, Lewin A, Patel S, et al. Combination of empagliflozin and linagliptin as second-line therapy in subjects with type 2 diabetes inadequately controlled on metformin. *Diabetes Care* 2015; 38(3): 384-393.

30 Lewin A, DeFronzo RA, Patel S, et al. Initial combination of empagliflozin and linagliptin in subjects with type 2 diabetes. *Diabetes Care* 2015; 38(3):394-402.

31 Owens DR, Swallow R, Dugi KA, Woerle HJ. Efficacy and safety of linagliptin in persons with type 2 diabetes inadequately controlled by a combination of metformin and sulphonylurea: a 24-week randomized study. *Diabet Med* 2011; 28(11): 1352-1361.

32 Taskinen MR, Rosenstock J, Tamminen I, et al. Safety and efficacy of linagliptin as add-on therapy to metformin in patients with type 2 diabetes: a randomized, double-blind, placebo-controlled study. *Diabetes Obes Metab* 2011; 13(1): 65-74.

33 Barnett AH, Charbonnel B, Li J, Donovan M, Fleming D, Iqbal N. Saxagliptin add-on therapy to insulin with or without metformin for type 2 diabetes mellitus: 52-week safety and efficacy. *Clin Drug Investig* 2013; 33(10): 707-717.

34 Barnett AH, Charbonnel B, Donovan M, Fleming D, Chen R. Effect of saxagliptin as add-on therapy in patients with poorly controlled type 2 diabetes on insulin alone or insulin combined with metformin. *Curr Med Res Opin* 2012; 28(4): 513-523.

35 Pan CY, Yang W, Tou C, Gause-Nilsson I, Zhao J. Efficacy and safety of saxagliptin in drug-naive Asian patients with type 2 diabetes mellitus: a randomized controlled trial. *Diabetes Metab Res Rev* 2012; 28(3): 268-275.

36 Yang W, Pan CY, Tou C, Zhao J, Gause-Nilsson I. Efficacy and safety of saxagliptin added to metformin in Asian people with type 2 diabetes mellitus: a randomized controlled trial. *Diabetes Res Clin Pract* 2011; 94(2): 217-224.

37 DeFronzo RA, Hissa MN, Garber AJ, et al. The efficacy and safety of saxagliptin when added to metformin therapy in patients with inadequately controlled type 2 diabetes with metformin alone. *Diabetes Care* 2009; 32(9): 1649-1655.

38 Hollander PL, Li J, Frederich R, Allen E, Chen R. Safety and efficacy of saxagliptin added to thiazolidinedione over 76 weeks in patients with type 2 diabetes mellitus. *Diab Vasc Dis Res* 2011; 8(2): 125-135.

39 Hollander P, Li J, Allen E, Chen R. Saxagliptin added to a thiazolidinedione improves glycemic control in patients with type 2 diabetes and inadequate control on thiazolidinedione alone. *J Clin Endocrinol Metab* 2009; 94(12): 4810-4819.

40 Pfutzner A, Paz-Pacheco E, Allen E, Frederich R, Chen R. Initial combination therapy with saxagliptin and metformin provides sustained glycaemic control and is well tolerated for up to 76 weeks. *Diabetes Obes Metab* 2011; 13(6): 567-576.

41 Jadzinsky M, Pfutzner A, Paz-Pacheco E, Xu Z, Allen E, Chen R. Saxagliptin given in combination with metformin as initial therapy improves glycaemic control in patients with type 2 diabetes compared with either monotherapy: a randomized controlled trial. *Diabetes Obes Metab* 2009; 11(6): 611-622.

42 Nowicki M, Rychlik I, Haller H, et al. Long-term treatment with the dipeptidyl peptidase-4 inhibitor saxagliptin in patients with type 2 diabetes mellitus and renal impairment: a randomised controlled 52-week efficacy and safety study. *Int J Clin Pract* 2011; 65(12): 1230-1239.

43 Roden M, Merker L, Christiansen AV, et al. Safety, tolerability and effects on cardiometabolic risk factors of empagliflozin monotherapy in drug-naive patients with type 2 diabetes: a double-blind extension of a Phase III randomized controlled trial. *Cardiovasc Diabetol* 2015; 14: 154.

44 Leiter LA, Carr MC, Stewart M, et al. Efficacy and safety of the once-weekly GLP-1 receptor agonist albiglutide versus sitagliptin in patients with type 2 diabetes and renal impairment: a randomized phase III study. *Diabetes Care* 2014; 37(10): 2723-2730.

45 Weinstock RS, Guerci B, Umpierrez G, Nauck MA, Skrivanek Z, Milicevic Z. Safety and efficacy of once-weekly dulaglutide versus sitagliptin after 2 years in metformin-treated patients with type 2 diabetes (AWARD-5): a randomized, phase III study. *Diabetes Obes Metab* 2015; 17(9): 849-858.

46 Nauck M, Weinstock RS, Umpierrez GE, Guerci B, Skrivanek Z, Milicevic Z. Efficacy and safety of dulaglutide versus sitagliptin after 52 weeks in type 2 diabetes in a randomized controlled trial (AWARD-5). *Diabetes Care* 2014; 37(8): 2149-2158.

47 Schernthaner G, Gross JL, Rosenstock J, et al. Canagliflozin compared with sitagliptin for patients with type 2 diabetes who do not have adequate glycemic control with metformin plus sulfonylurea: a 52-week randomized trial. *Diabetes Care* 2013; 36(9): 2508-2515.

48 Roden M, Weng J, Eilbracht J, et al. Empagliflozin monotherapy with sitagliptin as an active comparator in patients with type 2 diabetes: a randomised, double-blind, placebo-controlled, phase 3 trial. *Lancet Diabetes Endocrinol* 2013; 1(3): 208-219.

49 Dobs AS, Goldstein BJ, Aschner P, et al. Efficacy and safety of sitagliptin added to ongoing metformin and rosiglitazone combination therapy in a randomized placebo-controlled 54-week trial in patients with type 2 diabetes. *J Diabetes* 2013; 5(1): 68-79.

50 Fonseca V, Staels B, Morgan JN, et al. Efficacy and safety of sitagliptin added to ongoing metformin and pioglitazone combination therapy in a randomized, placebo-controlled, 26-week trial in patients with type 2 diabetes. *J Diabetes Complications* 2013; 27(2): 177-183.

51 Pratley R, Nauck M, Bailey T, et al. One year of liraglutide treatment offers sustained and more effective glycaemic control and weight reduction compared with sitagliptin, both in combination with metformin, in patients with type 2 diabetes: a randomised, parallel-group, open-label trial. *Int J Clin Pract* 2011; 65(4): 397-407.

52 Pratley RE, Nauck M, Bailey T, et al. Liraglutide versus sitagliptin for patients with type 2 diabetes who did not have adequate glycaemic control with metformin: a 26-week, randomised, parallel-group, open-label trial. *Lancet* 2010; 375(9724): 1447-1456.

53 Bergenstal RM, Wysham C, Macconell L, et al. Efficacy and safety of exenatide once weekly versus sitagliptin or pioglitazone as an adjunct to metformin for treatment of type 2 diabetes (DURATION-2): a randomised trial. *Lancet* 2010; 376(9739): 431-439.

54 Vilsboll T, Rosenstock J, Yki-Jarvinen H, et al. Efficacy and safety of sitagliptin when added to insulin therapy in patients with type 2 diabetes. *Diabetes Obes Metab* 2010; 12(2): 167-177.

55 Raz I, Chen Y, Wu M, et al. Efficacy and safety of sitagliptin added to ongoing metformin therapy in patients with type 2 diabetes. *Curr Med Res Opin* 2008; 24(2): 537-550.

56 Williams-Herman D, Johnson J, Teng R, et al. Efficacy and safety of sitagliptin and metformin as initial combination therapy and as monotherapy over 2 years in patients with type 2 diabetes. *Diabetes Obes Metab* 2010; 12(5): 442-451.

57 Williams-Herman D, Johnson J, Teng R, et al. Efficacy and safety of initial combination therapy with sitagliptin and metformin in patients with type 2 diabetes: a 54-week study. *Curr Med Res Opin* 2009; 25(3): 569-583.

58 Ahren B, Johnson SL, Stewart M, et al. HARMONY 3: 104-week randomized, double-blind, placebo- and active-controlled trial assessing the efficacy and safety of albiglutide compared with placebo, sitagliptin, and glimepiride in patients with type 2 diabetes taking metformin. *Diabetes Care* 2014; 37(8): 2141-2148.

59 Lavalle-Gonzalez FJ, Januszewicz A, Davidson J, et al. Efficacy and safety of canagliflozin compared with placebo and sitagliptin in patients with type 2 diabetes on background metformin monotherapy: a randomised trial. *Diabetologia* 2013; 56(12): 2582-2592.

60 Yoon KH, Shockey GR, Teng R, et al. Effect of initial combination therapy with sitagliptin, a dipeptidyl peptidase-4 inhibitor, and pioglitazone on glycemic control and measures of beta-cell function in patients with type 2 diabetes. *Int J Clin Pract* 2011; 65(2): 154-164.

61 Hermansen K, Kipnes M, Luo E, Fanurik D, Khatami H, Stein P. Efficacy and safety of the dipeptidyl peptidase-4 inhibitor, sitagliptin, in patients with type 2 diabetes mellitus inadequately controlled on glimepiride alone or on glimepiride and metformin. *Diabetes Obes Metab* 2007; 9(5): 733-745.

62 Ferrannini E, Berk A, Hantel S, et al. Long-term safety and efficacy of empagliflozin, sitagliptin, and metformin: an active-controlled, parallel-group, randomized, 78-week open-label extension study in patients with type 2 diabetes. *Diabetes Care* 2013; 36(12): 4015-4021.

63 Yang W, Xing X, Lv X, et al. Vildagliptin added to sulfonylurea improves glycemic control without hypoglycemia and weight gain in Chinese patients with type 2 diabetes mellitus. *J Diabetes* 2015; 7(2): 174-181.

64 Strain WD, Lukashevich V, Kothny W, Hoellinger MJ, Paldanius PM. Individualised treatment targets for elderly patients with type 2 diabetes using vildagliptin add-on or lone therapy (INTERVAL): a 24 week, randomised, double-blind, placebo-controlled study. *Lancet* 2013; 382(9890): 409-416.

65 Pan C, Xing X, Han P, et al. Efficacy and tolerability of vildagliptin as add-on therapy to metformin in Chinese patients with type 2 diabetes mellitus. *Diabetes Obes Metab* 2012; 14(8): 737-744.

66 Bosi E, Camisasca RP, Collober C, Rochotte E, Garber AJ. Effects of vildagliptin on glucose control over 24 weeks in patients with type 2 diabetes inadequately controlled with metformin. *Diabetes Care* 2007; 30(4): 890-895.

67 Fonseca V, Schweizer A, Albrecht D, Baron MA, Chang I, Dejager S. Addition of vildagliptin to insulin improves glycaemic control in type 2 diabetes. *Diabetologia* 2007; 50(6): 1148-1155.

68 Kothny W, Shao Q, Groop PH, Lukashevich V. One-year safety, tolerability and efficacy of vildagliptin in patients with type 2 diabetes and moderate or severe renal impairment. *Diabetes Obes Metab* 2012; 14(11): 1032-1039.

69 Lukashevich V, Schweizer A, Shao Q, Groop PH, Kothny W. Safety and efficacy of vildagliptin versus placebo in patients with type 2 diabetes and moderate or severe renal impairment: a prospective 24-week randomized placebo-controlled trial. *Diabetes Obes Metab* 2011; 13(10): 947-954.

70 Nauck MA, Stewart MW, Perkins C, et al. Efficacy and safety of once-weekly GLP-1 receptor agonist albiglutide (HARMONY 2): 52 week primary endpoint results from a randomised, placebo-controlled trial in patients with type 2 diabetes mellitus inadequately controlled with diet and exercise. *Diabetologia* 2016; 59(2): 266-274.

71 Home PD, Shamanna P, Stewart M, et al. Efficacy and tolerability of albiglutide versus placebo or pioglitazone over 1 year in people with type 2 diabetes currently taking metformin and glimepiride: HARMONY 5. *Diabetes Obes Metab* 2015; 17(2): 179-187.

72 Reusch J, Stewart MW, Perkins CM, et al. Efficacy and safety of once-weekly glucagon-like peptide 1 receptor agonist albiglutide (HARMONY 1 trial): 52-week primary endpoint results from a randomized, double-blind, placebo-controlled trial in patients with type 2 diabetes mellitus not controlled on pioglitazone, with or without metformin. *Diabetes Obes Metab* 2014; 16(12): 1257-1264.

73 Ferdinand KC, White WB, Calhoun DA, et al. Effects of the once-weekly glucagon-like peptide-1 receptor agonist dulaglutide on ambulatory blood pressure and heart rate in patients with type 2 diabetes mellitus. *Hypertension* 2014; 64(4): 731-737.

74 Wysham C, Blevins T, Arakaki R, et al. Efficacy and safety of dulaglutide added onto pioglitazone and metformin versus exenatide in type 2 diabetes in a randomized controlled trial (AWARD-1). *Diabetes Care* 2014; 37(8): 2159-2167.

75 Buse JB, Han J, Miller S, MacConell L, Pencek R, Wintle M. Addition of exenatide BID to insulin glargine: a post-hoc analysis of the effect on glycemia and weight across a range of insulin titration. *Curr Med Res Opin* 2014; 30(7): 1209-1218.

76 Buse JB, Bergenstal RM, Glass LC, et al. Use of twice-daily exenatide in Basal insulin-treated patients with type 2 diabetes: a randomized, controlled trial. *Ann Intern Med* 2011; 154(2): 103-112.

77 Liutkus J, Rosas GJ, Norwood P, et al. A placebo-controlled trial of exenatide twice-daily added to thiazolidinediones alone or in combination with metformin. *Diabetes Obes Metab* 2010; 12(12): 1058-1065.

78 Buse JB, Henry RR, Han J, Kim DD, Fineman MS, Baron AD. Effects of exenatide (exendin-4) on glycemic control over 30 weeks in sulfonylurea-treated patients with type 2 diabetes. *Diabetes Care* 2004; 27(11): 2628-2635.

79 Davies MJ, Bergenstal R, Bode B, et al. Efficacy of Liraglutide for Weight Loss Among Patients With Type 2 Diabetes: The SCALE Diabetes Randomized Clinical Trial. *JAMA* 2015; 314(7): 687-699.

80 Rosenstock J, Hanefeld M, Shamanna P, et al. Beneficial effects of once-daily lixisenatide on overall and postprandial glycemic levels without significant excess of hypoglycemia in type 2 diabetes inadequately controlled on a sulfonylurea with or without metformin (GetGoal-S). *J Diabetes Complications* 2014; 28(3): 386-392.

81 Yu PC, Han P, Liu X, et al. Lixisenatide treatment improves glycaemic control in Asian patients with type 2 diabetes mellitus inadequately controlled on metformin with or without sulfonylurea: a randomized, double-blind, placebo-controlled, 24-week trial (GetGoal-M-Asia). *Diabetes Metab Res Rev* 2014; 30(8): 726-735.

82 Pinget M, Goldenberg R, Niemoeller E, Muehlen-Bartmer I, Guo H, Aronson R. Efficacy and safety of lixisenatide once daily versus placebo in type 2 diabetes insufficiently controlled on pioglitazone (GetGoal-P). *Diabetes Obes Metab* 2013; 15(11): 1000-1007.

83 Riddle MC, Aronson R, Home P, et al. Adding once-daily lixisenatide for type 2 diabetes inadequately controlled by established basal insulin: a 24-week, randomized, placebo-controlled comparison (GetGoal-L). *Diabetes Care* 2013; 36(9): 2489-2496.

84 Riddle MC, Forst T, Aronson R, et al. Adding once-daily lixisenatide for type 2 diabetes inadequately controlled with newly initiated and continuously titrated basal insulin glargine: a 24-week, randomized, placebo-controlled study (GetGoal-Duo 1). *Diabetes Care* 2013; 36(9): 2497-2503.

85 Seino Y, Min KW, Niemoeller E, Takami A. Randomized, double-blind, placebo-controlled trial of the once-daily GLP-1 receptor agonist lixisenatide in Asian patients with type 2 diabetes insufficiently controlled on basal insulin with or without a sulfonylurea (GetGoal-L-Asia). *Diabetes Obes Metab* 2012; 14(10): 910-917.

86 Bolli GB, Munteanu M, Dotsenko S, et al. Efficacy and safety of lixisenatide once daily vs. placebo in people with Type 2 diabetes insufficiently controlled on metformin (GetGoal-F1). *Diabet Med* 2014; 31(2): 176-184.

87 Bode B, Stenlof K, Harris S, et al. Long-term efficacy and safety of canagliflozin over 104 weeks in patients aged 55-80 years with type 2 diabetes. *Diabetes Obes Meta*b 2015; 17(3): 294-303.

88 Bode B, Stenlof K, Sullivan D, Fung A, Usiskin K. Efficacy and safety of canagliflozin treatment in older subjects with type 2 diabetes mellitus: a randomized trial. *Hosp Pract (1995)* 2013; 41(2): 72-84.

89 Stenlof K, Cefalu WT, Kim KA, et al. Long-term efficacy and safety of canagliflozin monotherapy in patients with type 2 diabetes inadequately controlled with diet and exercise: findings from the 52-week CANTATA-M study. *Curr Med Res Opin* 2014; 30(2): 163-175.

90 Stenlof K, Cefalu WT, Kim KA, et al. Efficacy and safety of canagliflozin monotherapy in subjects with type 2 diabetes mellitus inadequately controlled with diet and exercise. *Diabetes Obes Metab* 2013; 15(4): 372-382.

91 Yale JF, Bakris G, Cariou B, et al. Efficacy and safety of canagliflozin over 52 weeks in patients with type 2 diabetes mellitus and chronic kidney disease. *Diabetes Obes Metab* 2014; 16(10): 1016-1027.

92 Yale JF, Bakris G, Cariou B, et al. Efficacy and safety of canagliflozin in subjects with type 2 diabetes and chronic kidney disease. *Diabetes Obes Metab* 2013; 15(5): 463-473.

93 Wilding JP, Charpentier G, Hollander P, et al. Efficacy and safety of canagliflozin in patients with type 2 diabetes mellitus inadequately controlled with metformin and sulphonylurea: a randomised trial. *Int J Clin Pract* 2013; 67(12): 1267-1282.

94 Forst T, Guthrie R, Goldenberg R, et al. Efficacy and safety of canagliflozin over 52 weeks in patients with type 2 diabetes on background metformin and pioglitazone. *Diabetes Obes Metab* 2014; 16(5): 467-477.

95 Bailey CJ, Morales VE, Woo V, Tang W, Ptaszynska A, List JF. Efficacy and safety of dapagliflozin monotherapy in people with Type 2 diabetes: a randomized double-blind placebo-controlled 102-week trial. *Diabet Med* 2015; 32(4): 531-541.

96 Ferrannini E, Ramos SJ, Salsali A, Tang W, List JF. Dapagliflozin monotherapy in type 2 diabetic patients with inadequate glycemic control by diet and exercise: a randomized, double-blind, placebo-controlled, phase 3 trial. *Diabetes Care* 2010; 33(10): 2217-2224.

97 Cefalu WT, Leiter LA, de Bruin TW, Gause-Nilsson I, Sugg J, Parikh SJ. Dapagliflozin's Effects on Glycemia and Cardiovascular Risk Factors in High-Risk Patients With Type 2 Diabetes: A 24-Week, Multicenter, Randomized, Double-Blind, Placebo-Controlled Study With a 28-Week Extension. *Diabetes Care* 2015; 38(7): 1218-1227.

98 Jabbour SA, Hardy E, Sugg J, Parikh S. Dapagliflozin is effective as add-on therapy to sitagliptin with or without metformin: a 24-week, multicenter, randomized, double-blind, placebo-controlled study. *Diabetes Care* 2014; 37(3): 740-750.

99 Kohan DE, Fioretto P, Tang W, List JF. Long-term study of patients with type 2 diabetes and moderate renal impairment shows that dapagliflozin reduces weight and blood pressure but does not improve glycemic control. *Kidney Int* 2014; 85(4): 962-971.

100 Leiter LA, Cefalu WT, de Bruin TW, Gause-Nilsson I, Sugg J, Parikh SJ. Dapagliflozin added to usual care in individuals with type 2 diabetes mellitus with preexisting cardiovascular disease: a 24-week, multicenter, randomized, double-blind, placebo-controlled study with a 28-week extension. *J Am Geriatr Soc* 2014; 62(7): 1252-1262.

101 Wilding JP, Woo V, Rohwedder K, Sugg J, Parikh S. Dapagliflozin in patients with type 2 diabetes receiving high doses of insulin: efficacy and safety over 2 years. *Diabetes Obes Metab* 2014; 16(2): 124-136.

102 Wilding JP, Woo V, Soler NG, et al. Long-term efficacy of dapagliflozin in patients with type 2 diabetes mellitus receiving high doses of insulin: a randomized trial. *Ann Intern Med* 2012; 156(6): 405-415.

103 Bailey CJ, Gross JL, Hennicken D, Iqbal N, Mansfield TA, List JF. Dapagliflozin add-on to metformin in type 2 diabetes inadequately controlled with metformin: a randomized, double-blind, placebo-controlled 102-week trial. *BMC Med* 2013; 11: 43.

104 Bailey CJ, Iqbal N, T'Joen C, List JF. Dapagliflozin monotherapy in drug-naive patients with diabetes: a randomized-controlled trial of low-dose range. *Diabetes Obes Metab* 2012; 14(10): 951-959.

105 Ljunggren O, Bolinder J, Johansson L, et al. Dapagliflozin has no effect on markers of bone formation and resorption or bone mineral density in patients with inadequately controlled type 2 diabetes mellitus on metformin. *Diabetes Obes Metab* 2012; 14(11): 990-999.

106 Rosenstock J, Vico M, Wei L, Salsali A, List JF. Effects of dapagliflozin, an SGLT2 inhibitor, on HbA(1c), body weight, and hypoglycemia risk in patients with type 2 diabetes inadequately controlled on pioglitazone monotherapy. *Diabetes Care* 2012; 35(7): 1473-1478.

107 Strojek K, Yoon KH, Hruba V, Elze M, Langkilde AM, Parikh S. Effect of dapagliflozin in patients with type 2 diabetes who have inadequate glycaemic control with glimepiride: a randomized, 24-week, double-blind, placebo-controlled trial. *Diabetes Obes Metab* 2011; 13(10): 928-938.

108 Kovacs CS, Seshiah V, Merker L, et al. Empagliflozin as Add-on Therapy to Pioglitazone With or Without Metformin in Patients With Type 2 Diabetes Mellitus. *Clin Ther* 2015; 37(8): 1773-1788.

109 Barnett AH, Mithal A, Manassie J, et al. Efficacy and safety of empagliflozin added to existing antidiabetes treatment in patients with type 2 diabetes and chronic kidney disease: a randomised, double-blind, placebo-controlled trial. *Lancet Diabetes Endocrinol* 2014; 2(5): 369-384.

110 Rosenstock J, Jelaska A, Frappin G, et al. Improved glucose control with weight loss, lower insulin doses, and no increased hypoglycemia with empagliflozin added to titrated multiple daily injections of insulin in obese inadequately controlled type 2 diabetes. *Diabetes Care* 2014; 37(7): 1815-1823.

111 DeFronzo RA, Ratner RE, Han J, Kim DD, Fineman MS, Baron AD. Effects of exenatide (exendin-4) on glycemic control and weight over 30 weeks in metformin-treated patients with type 2 diabetes. *Diabetes Care* 2005; 28(5): 1092-100.

112 ClinicalTrials.gov Identifier: NCT00603239. <https://clinicaltrials.gov/ct2/show/NCT00603239> (accessed November 20, 2018).

113 ClinicalTrials.gov Identifier: NCT00701935. <https://clinicaltrials.gov/ct2/show/NCT00701935> (accessed November 20, 2018).

114 Buse JB, Henry RR, Han J, Kim DD, Fineman MS, Baron AD. Effects of exenatide (exendin-4) on glycemic control over 30 weeks in sulfonylurea-treated patients with type 2 diabetes. *Diabetes Care* 2004; 27(11): 2628-35.

115 Marre M, Shaw J, Brändle M, et al. Liraglutide, a once-daily human GLP-1 analogue, added to a sulphonylurea over 26 weeks produces greater improvements in glycaemic and weight control compared with adding rosiglitazone or placebo in subjects with Type 2 diabetes (LEAD-1 SU). *Diabet Med* 2009; 26(3): 268-78.

116 Russell-Jones D, Vaag A, Schmitz O, et al. Liraglutide vs insulin glargine and placebo in combination with metformin and sulfonylurea therapy in type 2 diabetes mellitus (LEAD-5 met+SU): a randomised controlled trial. *Diabetologia* 2009; 52(10): 2046-55.

117 Zinman B, Gerich J, Buse JB, et al. Efficacy and safety of the human glucagon-like peptide-1 analog liraglutide in combination with metformin and thiazolidinedione in patients with type 2 diabetes (LEAD-4 Met+TZD). *Diabetes Care* 2009; 32(7): 1224-30.

118 Raz I, Fonseca V, Kipnes M, et al. Efficacy and safety of taspoglutide monotherapy in drug-naive type 2 diabetic patients after 24 weeks of treatment: results of a randomized, double-blind, placebo-controlled phase 3 study (T-emerge 1). *Diabetes Care* 2012; 35(3): 485-7.

119 Russell-Jones D, Cuddihy RM, Hanefeld M, et al. Efficacy and safety of exenatide once weekly versus metformin, pioglitazone, and sitagliptin used as monotherapy in drug-naive patients with type 2 diabetes (DURATION-4): a 26-week double-blind study. *Diabetes Care* 2012; 35(2): 252-8.

120 Bergenstal R, Lewin A, Bailey T, Chang D, Gylvin T, Roberts V; NovoLog Mix-vs.-Exenatide Study GroupEfficacy and safety of biphasic insulin aspart 70/30 versus exenatide in subjects with type 2 diabetes failing to achieve glycemic control with metformin and a sulfonylurea. *Curr Med Res Opin* 2009; 25(1): 65-75.

121 Pratley R.E., Reusch J.E., Fleck P.R., Wilson C.A., Mekki Q. Efficacy and safety ofthe dipeptidyl peptidase-4 inhibitor alogliptin added to pioglitazone in patients withtype 2 diabetes: a randomized, double-blind, placebo-controlled study. *Curr MedRes Opin* 2009; 25: 2361-2371.

122 Del Prato S, Barnett AH, Huisman H, Neubacher D, Woerle HJ, Dugi KA. Effect oflinagliptin monotherapy on glycaemic control and markers of β-cell function in patients with inadequately controlled type 2 diabetes: a randomized controlled trial. *Diabetes Obes Metab* 2011; 13: 258-67.

123 Chacra AR, Tan GH, Ravichandran S, List J, Chen R. CV181040 Investigators.Safety and efficacy of saxagliptin in combination with submaximal sulphonylureaversus up-titrated sulphonylurea over 76 weeks. *Diab Vasc Dis Res* 2011; 8: 150-159.

124 ClinicalTrials.gov Identifier: NCT00316082. [https://clinicaltrials.gov/ct2/show/NCT00316082](https://clinicaltrials.gov/ct2/show/NCT00603239) (accessed November 20, 2018).

125 Chan J.C., Scott R., rjona Ferreira J.C. et al. Safety and efficacy of sitagliptin inpatients with type 2 diabetes and chronic renal insufficiency. *Diabetes Obes Metab* 2008; 10: 545-555.

126 Charbonnel B., Karasik A., Liu J., Wu M., Meininger G. Efficacy and safety of thedipeptidyl peptidase-4 inhibitor sitagliptin added to ongoing metformin therapy inpatients with type 2 diabetes inadequately controlled with metformin alone. *Diabetes Care* 2006; 29: 2638-2643.

127 Raz I., Hanefeld M., Xu L., Caria C., Williams-Herman D., Khatami H. Efficacy andsafety of the dipeptidyl peptidase-4 inhibitor sitagliptin as monotherapy in patientswith type 2 diabetes mellitus. *Diabetologia* 2006; 49: 2564-2571.

128 ClinicalTrials.gov Identifier: NCT00482729. <https://clinicaltrials.gov/ct2/show/NCT00482729> (accessed on 18 November, 2018).

129 ClinicalTrials.gov Identifier: NCT00813995. <https://clinicaltrials.gov/ct2/show/NCT00813995> (accessed on 18 November, 2018).

130 Barzilai N, Guo H, Mahoney EM, et al. Efficacy and tolerability of sitagliptin monotherapy in elderly patients with type 2 diabetes: a randomized, double-blind, placebo-controlled trial. *Curr Med Res Opin* 2011; 27: 1049-1058.

131 Foley JE, Sreenan S. Efficacy and safety comparison between the DPP-4 inhibitorvildagliptin and the sulfonylurea gliclazide after two years of monotherapy in drug-naïve patients with type 2 diabetes. *Horm Metab Res* 2009; 41: 905-909.

132 Dejager S., Razac S., Foley J.E., Schweizer A. Vildagliptin in drug-naive patients with type 2 diabetes: a 24-week, double-blind, randomized, placebo-controlled, multiple-dose study. *Horm Metab Res* 2007; 39: 218-223.

133 Scherbaum WA, Schweizer A, Mari A, et al. Efficacy and tolerability of vildagliptin in drug-naïve patients with type 2 diabetes and mild hyperglycaemia*. *Diabetes Obes Metab* 2008; 10: 675-682.

134 Thrasher J, Daniels K, Patel S, Whetteckey J, Woerle HJ. Efficacy and safety of linagliptin in black/African American patients with type 2 diabetes: A 6-month, randomized, double-blind, placebo-controlled study. *Endocrine Pract* 2014; 20(5): 412-420.

135 ClinicalTrials.gov Identifier: NCT00972244. <https://clinicaltrials.gov/ct2/show/results/NCT00972244> (accessed November 20, 2018).

136 Roden M, Merker L, EMPA-REG EXTEND™ MONO investigators, et al. Safety, tolerability and effects on cardiometabolic risk factors of empagliflozin monotherapy in drug-naïve patients with type 2 diabetes: a double-blind extension of a Phase III randomized controlled trial. *Cardiovasc Diabeto*l 2015; 14: 154.

137 ClinicalTrials.gov Identifier: NCT00289848. <https://clinicaltrials.gov/ct2/show/results/NCT00289848> (accessed November 20, 2018).

138 Gantz I, Chen M, Suryawanshi S, et al. A randomized, placebo-controlled study of the cardiovascular safety of the once-weekly DPP-4 inhibitor omarigliptin in patients with type 2 diabetes mellitus. *Cardiovasc Diabetol* 2017; 16(1): 112.

139 Dungan KM, Weitgasser R, Perez Manghi F, et al. A 24-week study to evaluate the efficacy and safety of once-weekly dulaglutide added on to glimepiride in type 2 diabetes (AWARD-8). *Diabetes Obes Metab* 2016; 18(5): 475-82.

140 Ferrannini E, Ramos SJ, Salsali A, Tang W, List JF. Dapagliflozin monotherapy in type 2 diabetic patients with inadequate glycemic control by diet and exercise: a randomized, double-blind, placebo-controlled, phase 3 trial. *Diabetes Care* 2010; 33(10): 2217-24.

141 Rosenstock J, Jelaska A, Zeller C, Kim G, Broedl UC, Woerle HJ; EMPA-REG BASALTM trial investigators. Impact of empagliflozin added on to basal insulin in type 2 diabetes inadequately controlled on basal insulin: a 78-week randomized, double-blind, placebo-controlled trial. *Diabetes Obes Metab* 2015; 17(10): 936-48.

142 Stenlöf K, Cefalu WT, Kim KA, et al. Long-term efficacy and safety of canagliflozin monotherapy in patients with type 2 diabetes inadequately controlled with diet and exercise: findings from the 52-week CANTATA-M study. *Curr Med Res Opin* 2014; 30(2): 163-75.

143 Henry RR, Murray AV, Marmolejo MH, Hennicken D, Ptaszynska A, List JF. Dapagliflozin, metformin XR, or both: initial pharmacotherapy for type 2 diabetes, a randomised controlled trial. *Int J Clin Pract* 2012y; 66(5): 446-56.

144 Kohan DE, Fioretto P, Tang W, List JF. Long-term study of patients with type 2 diabetes and moderate renal impairment shows that dapagliflozin reduces weight and blood pressure but does not improve glycemic control. *Kidney Int* 2014; 85(4): 962-71.

145 Tikkanen I, Narko K, Zeller C, Green A, Salsali A, Broedl UC, Woerle HJ; EMPA-REG BP Investigators. Empagliflozin reduces blood pressure in patients with type 2 diabetes and hypertension. *Diabetes Care* 2015; 38(3): 420-8.

146 ClinicalTrials.gov Identifier: NCT00972244. <https://clinicaltrials.gov/ct2/show/results/NCT00972244> (accessed November 20, 2018).

Supplementary Figures

**Fig.S1. Flow diagram of scientific literature search and study selection**


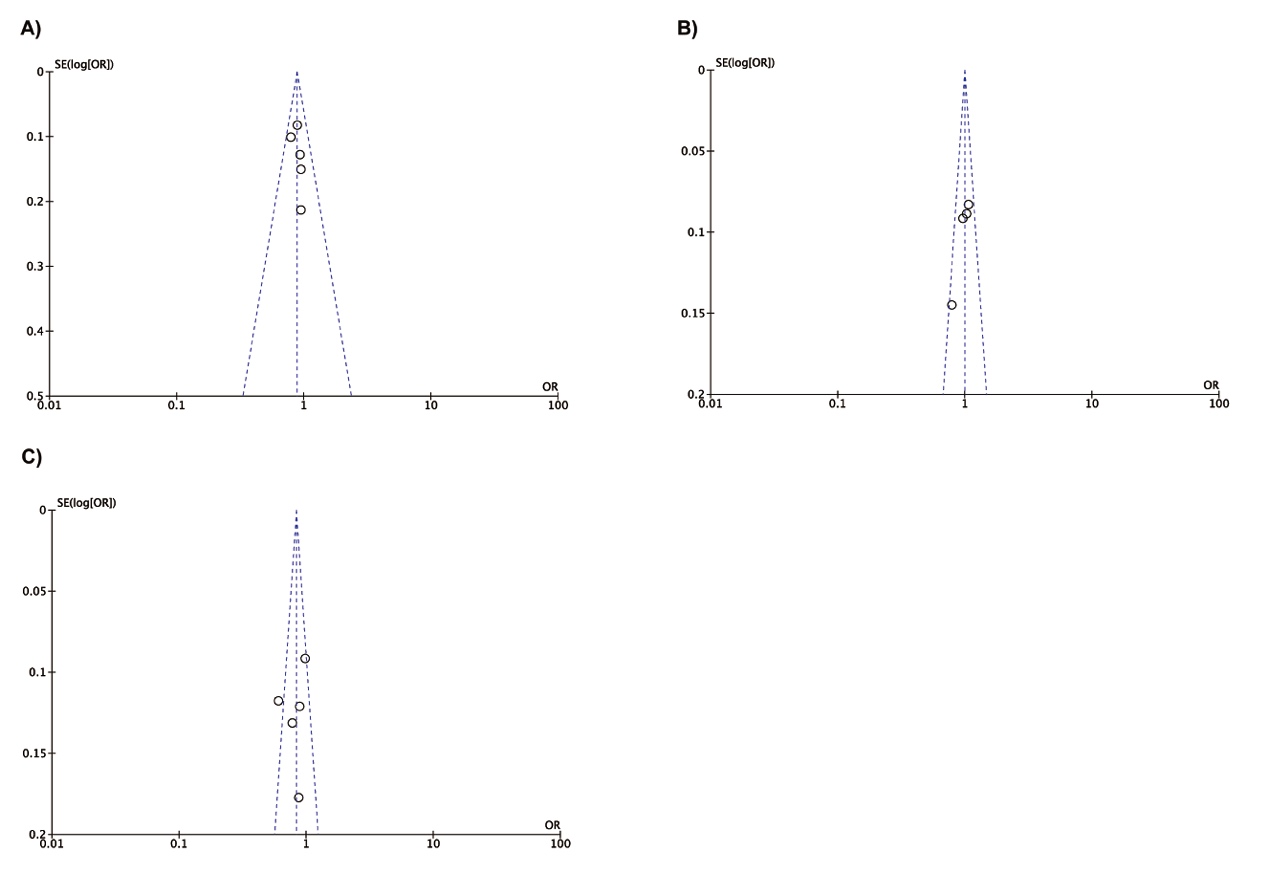


**Fig.S2. Funnel plot showing publication bias for the effect of antidiabetic drugs on cardiovascular mortality rate in type 2 diabetes mellitus patients**

(A) GLP-1 receptor agonists; (B) DPP-4 inhibitors; (C) SGLT-2 inhibitors.


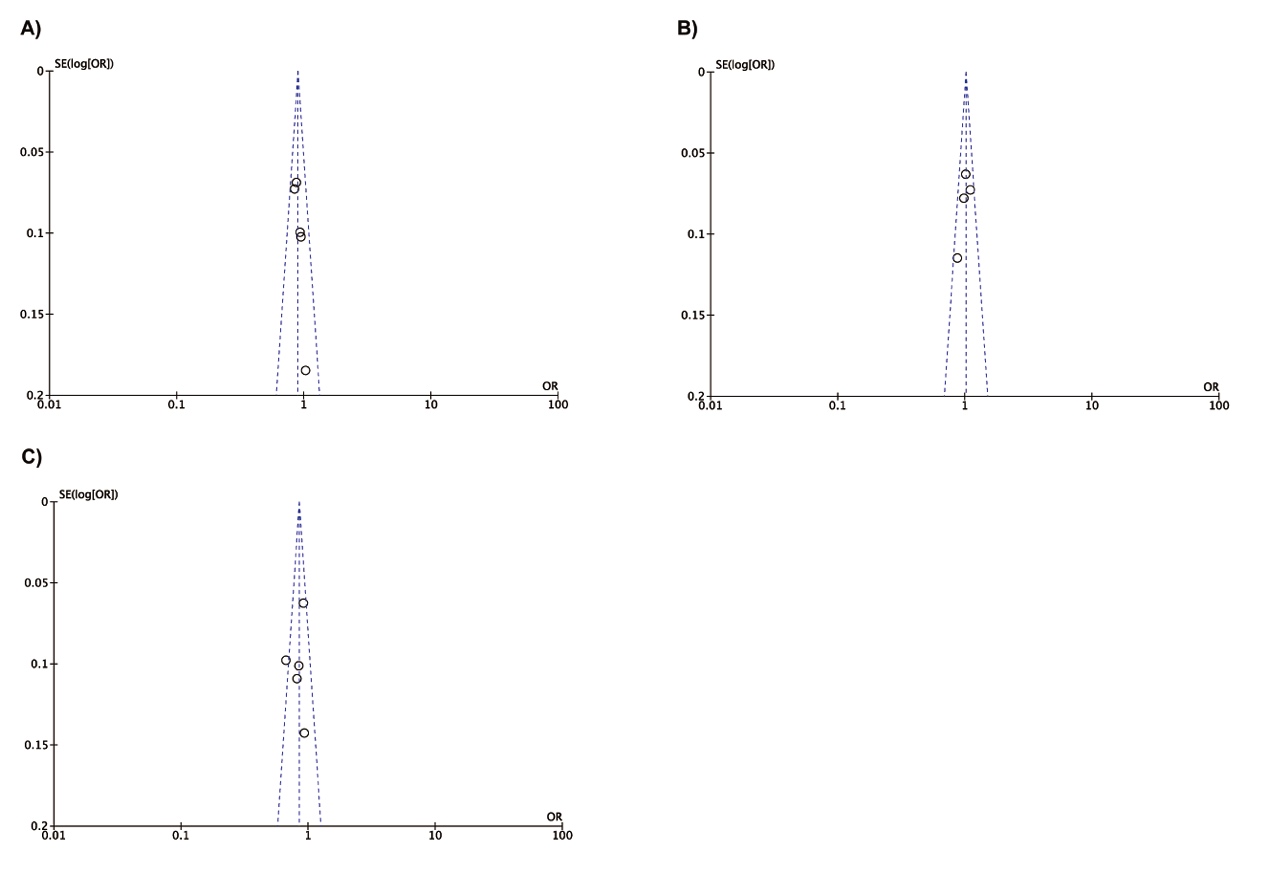


**Fig.S3. Funnel plot showing publication bias for the effect of antidiabetic drugs on all-cause mortality rate in type 2 diabetes mellitus patients**

(A) GLP-1 receptor agonists; (B) DPP-4 inhibitors; (C) SGLT-2 inhibitors.


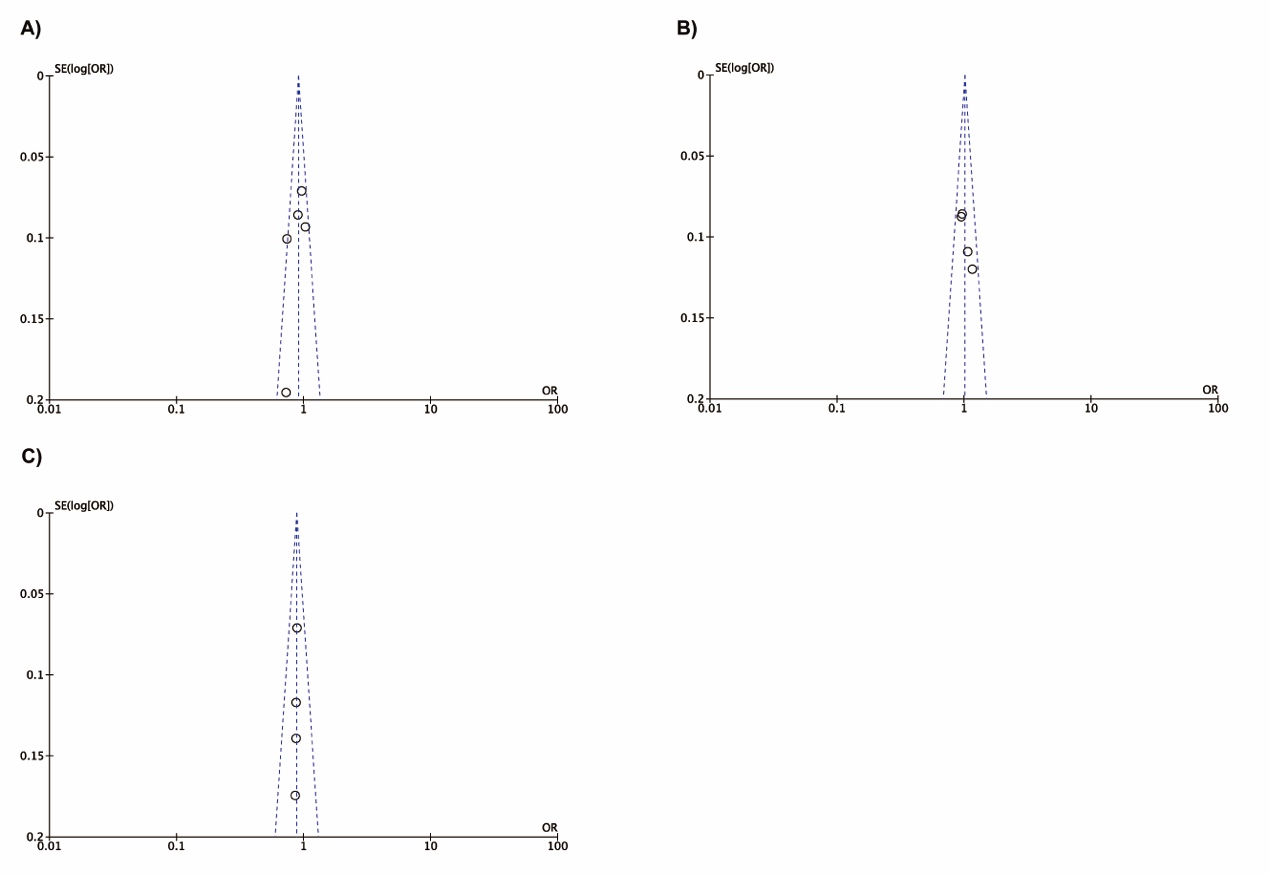


**Fig.S4. Funnel plot showing publication bias for the effect of antidiabetic drugs on frequencies of nonfatal myocardial infarction in type 2 diabetes mellitus patients**

(A) GLP-1 receptor agonists; (B) DPP-4 inhibitors; (C) SGLT-2 inhibitors.


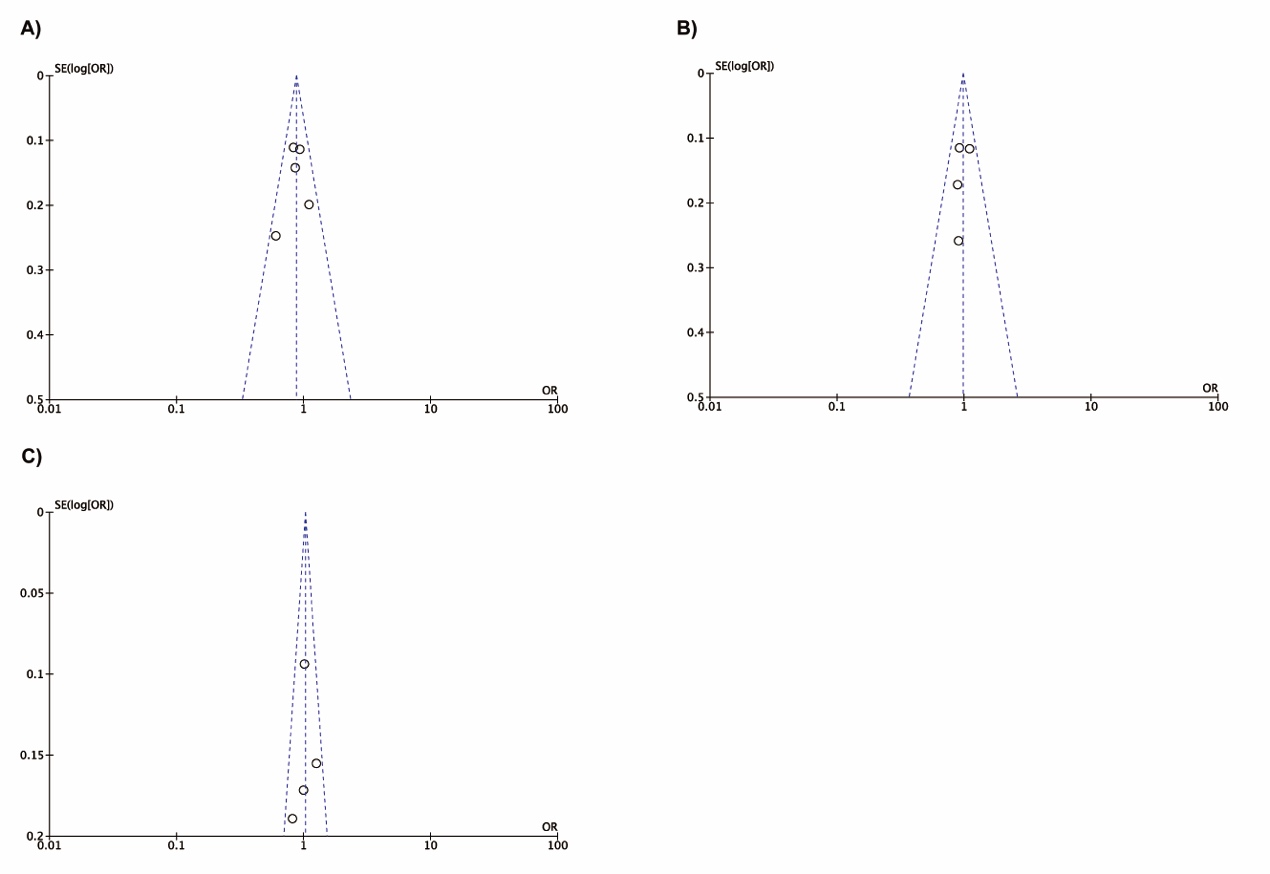


**Fig.S5. Funnel plot showing publication bias for the effect of antidiabetic drugs on frequencies of nonfatal stroke in type 2 diabetes mellitus patients**

(A) GLP-1 receptor agonists; (B) DPP-4 inhibitors; (C) SGLT-2 inhibitors.


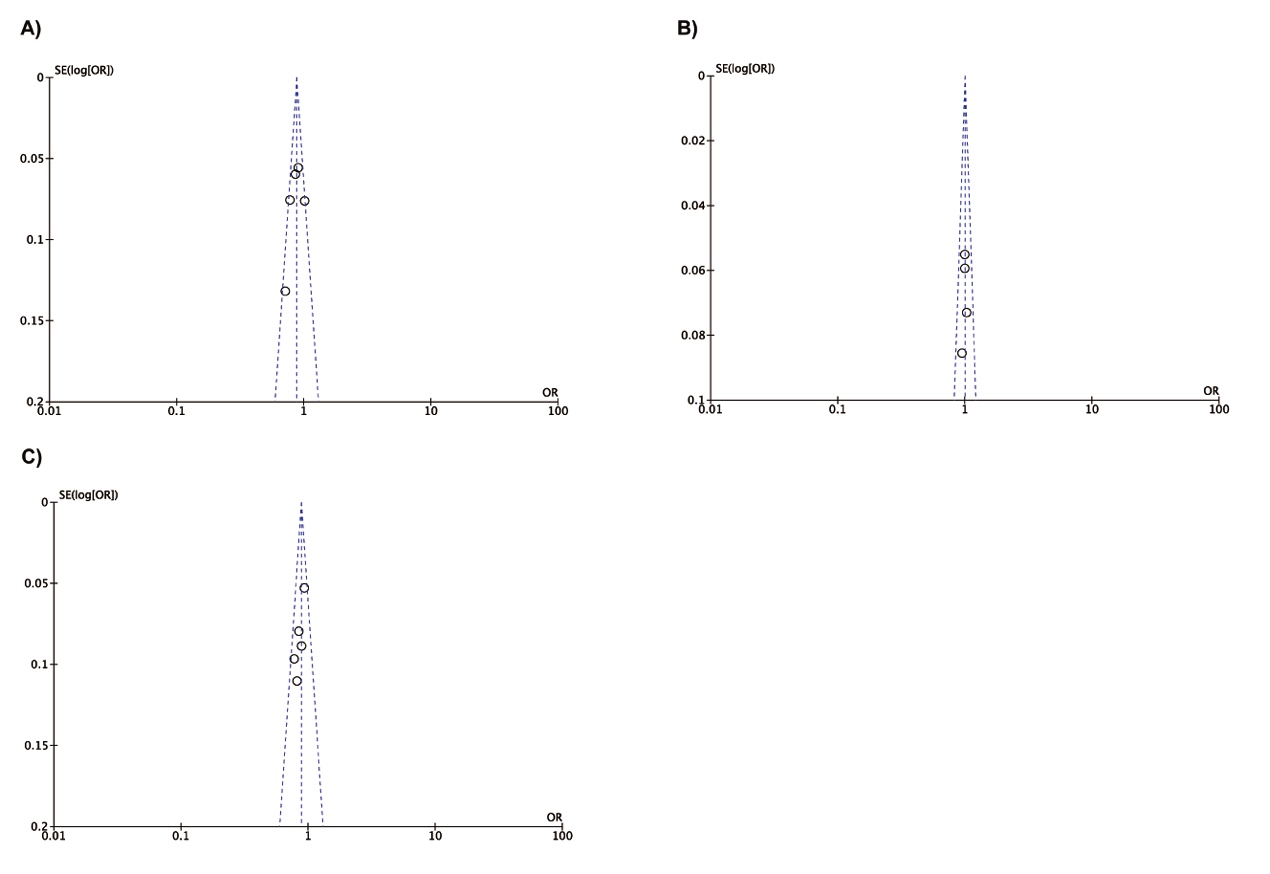


**Fig.S6. Funnel plot showing publication bias for the effect of antidiabetic drugs on frequencies of major adverse cardiovascular events (MACE) in type 2 diabetes mellitus patients**

(A) GLP-1 receptor agonists; (B) DPP-4 inhibitors; (C) SGLT-2 inhibitors.

MACE = major adverse cardiovascular events (defined as the composite of cardiovascular mortality, nonfatal myocardial infarction and nonfatal stroke).


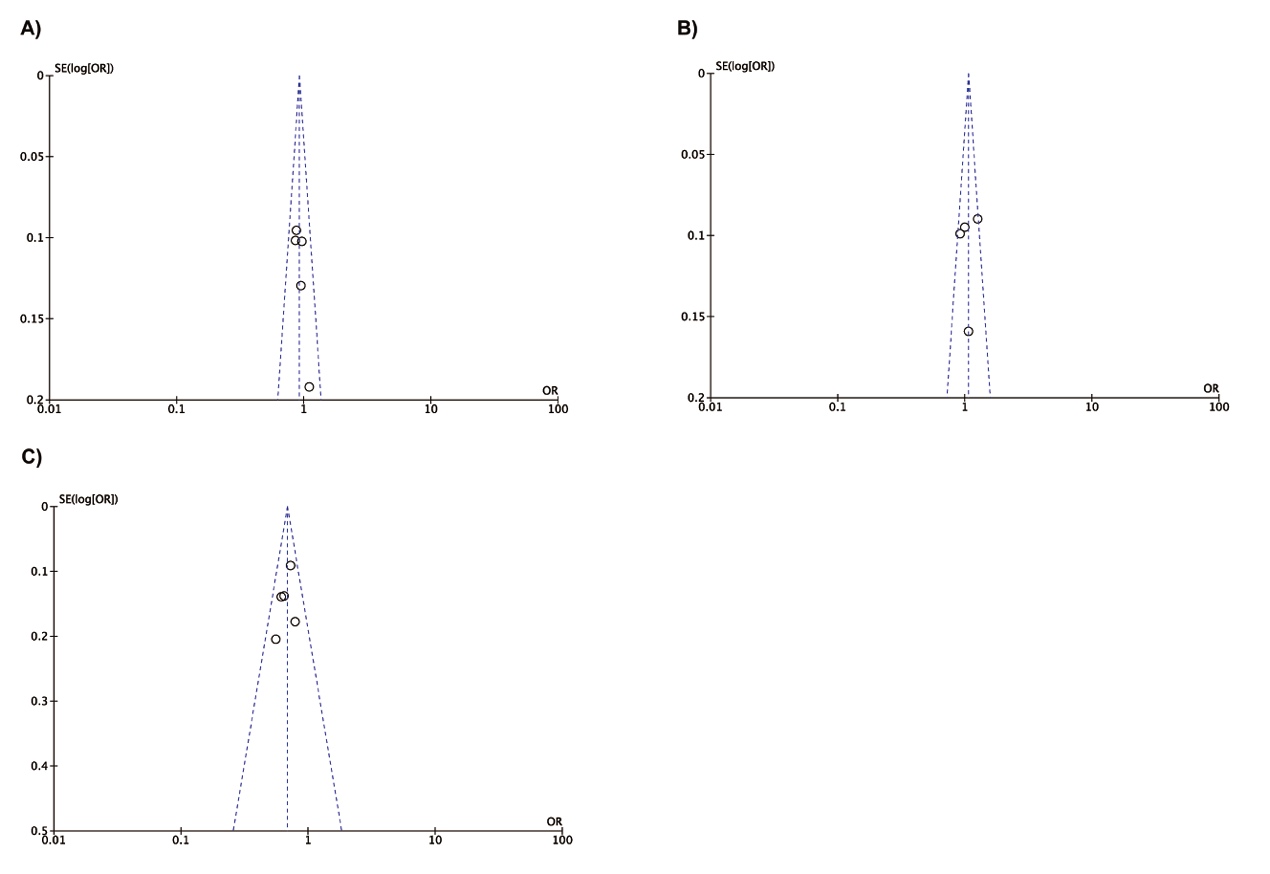


**Fig.S7. Funnel plot showing publication bias for the effect of antidiabetic drugs on frequencies of hospitalisation for heart failure in type 2 diabetes mellitus patients**

(A) GLP-1 receptor agonists; (B) DPP-4 inhibitors; (C) SGLT-2 inhibitors.


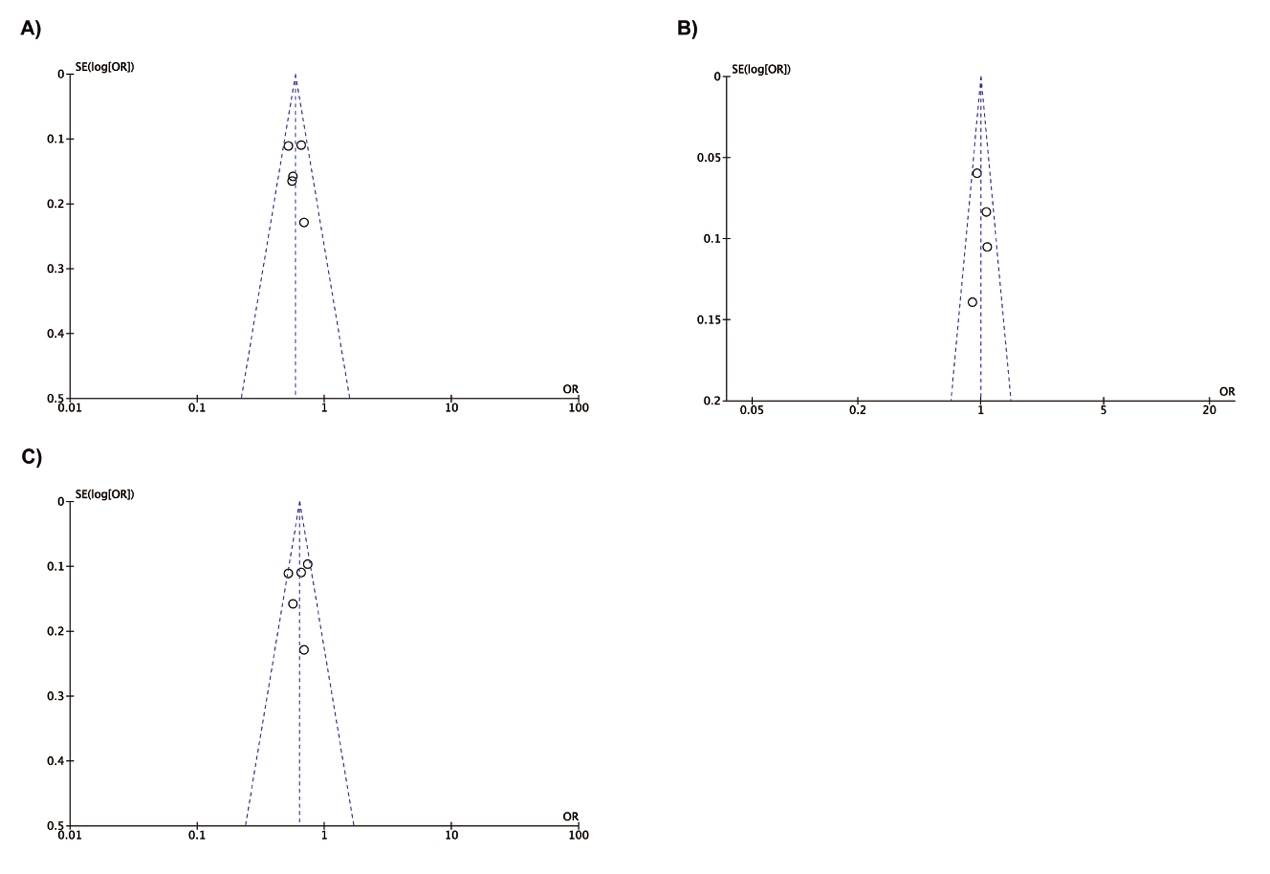


**Fig.S8. Funnel plot showing publication bias for the effect of antidiabetic drugs on frequencies of renal composite outcome in type 2 diabetes mellitus patients**

(A) GLP-1 receptor agonists; (B) DPP-4 inhibitors; (C) SGLT-2 inhibitors.
